# Identifying early-warning signals of critical transitions with strong noise by dynamical network markers

**DOI:** 10.1038/srep17501

**Published:** 2015-12-09

**Authors:** Rui Liu, Pei Chen, Kazuyuki Aihara, Luonan Chen

**Affiliations:** 1 School of Mathematics, South China University of Technology, Guangzhou 510640, China; 2 School of Computer Science and Engineering, South China University of Technology, Guangzhou 510640, China; 3 Collaborative Research Center for Innovative Mathematical Modelling, Institute of Industrial Science, University of Tokyo, Tokyo 153-8505, Japan; 4 Key Laboratory of Systems Biology, Innovation Center for Cell Signaling Network, Institute of Biochemistry and Cell Biology, Shanghai Institutes for Biological Sciences, Chinese Academy of Sciences, Shanghai 200031, China

## Abstract

Identifying early-warning signals of a critical transition for a complex system is difficult, especially when the target system is constantly perturbed by big noise, which makes the traditional methods fail due to the strong fluctuations of the observed data. In this work, we show that the critical transition is not traditional state-transition but probability distribution-transition when the noise is not sufficiently small, which, however, is a ubiquitous case in real systems. We present a model-free computational method to detect the warning signals before such transitions. The key idea behind is a strategy: “making big noise smaller” by a distribution-embedding scheme, which transforms the data from the observed state-variables with big noise to their distribution-variables with small noise, and thus makes the traditional criteria effective because of the significantly reduced fluctuations. Specifically, increasing the dimension of the observed data by moment expansion that changes the system from state-dynamics to probability distribution-dynamics, we derive new data in a higher-dimensional space but with much smaller noise. Then, we develop a criterion based on the dynamical network marker (DNM) to signal the impending critical transition using the transformed higher-dimensional data. We also demonstrate the effectiveness of our method in biological, ecological and financial systems.

Complex systems in ecology, biology, economics and many other fields often undergo slow changes affected by various external factors, whose persistent effects sometimes result in drastic or qualitative changes of system states from one stable state (i.e., the before-transition state) to another stable state (i.e., the after-transition state) through a pre-transition state (Fig. 1a, Fig. S3)^1^,^2^,^3^. For many natural and engineered systems, it is crucial to detect early-warning signals before this critical transition so as to prevent from or get ready for such a catastrophic event. Recent studies in dynamical systems theory show that critical slowing-down (CSD)^4^ can be used as a leading indicator to predict such sharp transitions, and has been applied to detect regime shifts or collapse in ecosystems^5^,^6^,^7^,^8^,^9^, climate systems^10^,^11^,^12^,^13^, biological systems^2^,^14^ and financial markets^15^,^16^. CSD-related research has become a hot topic and is increasingly attracting much attention from communities of both natural and social sciences. However, theoretically the signals based on CSD appear only when the system state approaches sufficiently near the bifurcation point or the tipping point (Fig. 1a), which implies that the CSD principle holds only for a system perturbed with small noise because the sharp transition of a system with big noise may occur far from the bifurcation point (Fig. 1b). In other words, the transition will emerge stochastically far before the deterministic bifurcation, and strong nonlinearities brought by the big noise will violate the assumptions of the CSD, i.e., a linear restoring force. Moreover, eigenvalues based analysis, e.g., spectral analysis, pseudospectra analysis and principle component analysis^17^,^18^,^19^,^20^, also fail in indicating the upcoming state change since signals from linear terms are highly disturbed by wild fluctuations and thus obscure, although pseudospectra analysis can provide the additional information on ill-conditioned cases. On the other hand, data observed from real-world systems such as ecosystems^21^,^22^, electric power systems^23^ and biomedical systems^24^,^25^, are usually intrinsically or extrinsically convoluted with big noise, for which the existing approaches may fail^26^.

There is a common feature for complex systems during the process of a state transition near a tipping point, that is, the dynamical process of a system along the time or parameter change can generally be expressed by the three states from the before-transition state through the pre-transition state to the after-transition state (Fig. S3 and Table S1)^27^: First, the before-transition state corresponds to an attractor like a stable equilibrium before the transition, during which the system undergoes changes gradually. Second, the pre-transition state is the critical state, which is actually the limit of the before-transition state just before the imminent drastic transition. A system in this state is easily affected by external perturbations and driven into another stable state, i.e., the after-transition state. In contrast, appropriate perturbations of system parameters also can pull the system back to the before-transition state. Third, the after-transition state is referred to an attractor like another stable equilibrium after the critical (or phase) transition, which is significantly different from the before-transition state and the pre-transition state.

As widely used in physics, critical slowing-down (CSD)^4^ for single variables has been considered as a leading indicator to predict such critical transitions provided that the system is fluctuated by small noise, which assumes the linear restoring force. Recently, based on dynamical systems theory, we developed a network-based criteria for multi-dimensional data, i.e., the dynamical network biomarker (DNB), to detect the pre-transition state of biomedical systems (e.g., complex diseases)^2^,^14^,^28^. For a general system, we further showed, when it approaches the pre-transition state, there exists at least one dominant group of variables among all variables, which are strongly correlated but wildly fluctuated. Specifically, when the system approaches the pre-transition state, we can prove that a dominant group of variables appear and satisfy the following three conditions^2^.

1. The standard deviation (SD) of each variable in the dominant group drastically increases.
2. The correlation (PCC_*in*_) for each pair of variables within the dominant group increases.
3. The correlation (PCC_*out*_) between one variable in the dominant group and another one outside decreases.

Actually, the three conditions above hold even if all variables in the system are the dominant group members. This feature at the pre-transition state also implies the weak resilience on the state dynamics.

Due to such collective dynamics at the critical state, this group of variables is expected to form a subnetwork or module from a network viewpoint and thus is also called the dynamical network marker (DNM) for general systems or dynamical network biomarker (DNB) for biological systems^27^, which characterizes the dynamical features of a general stochastic system with small noise near the tipping point. To sensitively signal the emergence of the critical transition, we adopt the following score from the above conditions and notations:


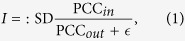


where 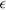 is a small positive constant to avoid zero division. Clearly, from the observed data with a few number of samples or time points, we can detect the early-warning signals of the state-transition for the multi-variable system by identifying its DNM with a maximal (or drastically increased) *I* provided that noise level is small (see Supplementary Information (SI) D for the details of the computational procedure). Generally, the critical point detected by DNM is near the bifurcation point of the corresponding deterministic system.

DNM provides a theoretical basis and a computational way for detecting the early-warning signals of the critical state-transition for multi-dimensional data with small noise (Table S1, Fig. S3). Based on the theoretical results of DNM, we know intuitively: (1) appearance of a group of collectively fluctuated variables among high-dimensional data implies the emergence of the critical transition; (2) identifying the after-transition state requires the differential information of state variables but predicting the transition (or after-transition state) further requires the differential interaction information among state variables, which means that identifying a state and predicting a state require different information. The effectiveness of DNM on several real biomedical systems has been successfully validated^2^,^28^,^29^,^30^, and the comparison with another multi-variable method is also shown in SI C.2. Note that, near the critical state, there may be multiple DNMs appearing, but we can provide the early-warning signals by detecting one of them (see A.1 in SI). Clearly, with synergetic effect of the three conditions, the score of DNM in (1) is expected to generate a strong signal even with a small number of samples. Note that CSD mainly characterizes the dynamics of single-variables, whereas DNM characterizes that of multi-variables or a network. Actually, for a single-variable system, DNM is equivalent to the principle of CSD.

In this study, based on dynamical systems theory we developed a probability distribution embedding scheme by converting state-dynamics with big noise into distribution-dynamics with small noise so that we can detect the early-warning signals before the critical regime shift in complex systems even with strong fluctuations or big noise. The key idea behind this method is to reduce the noise level by exploring the distribution-dynamics, and thus the traditional methods based on CSD can be directly applied to the transformed higher-dimensional data with smaller noise (Fig. 1). Specifically, transforming the observed data of the state by moment expansion, we can derive new data of the corresponding probability distribution in a higher-dimensional space but with much smaller noise (from Fig. 1b–e). Then, we further extend CSD for single variables to a dynamical network marker (DNM) for multi-variables or a network, and develop a criterion based on DNM to detect the early-warning signals of critical transitions in this transformed distribution-dynamics. We show that, by expanding the moments up to the 2nd order and thus increasing the dimension of the data from the original *n* to at most *n*(*n* + 3)/2, the fluctuations or noise levels are significantly reduced. In such a way, the original state system with big noise is transformed into a moment system with more variables representing the distribution of the state but with smaller noise. Thus, owing to the small noise, the CSD principle works well again. To further apply CSD to a multi-variable system in the transformed higher-dimensional space, DNM is developed to detect the early-warning signal for the critical transition (Fig. 1c,e), which clearly takes place much earlier than the bifurcation point of the original system.

Note that when the noise is not sufficiently small, the state-transition becomes nondeterministic or stochastic, i.e., the critical transition results not from local state-dynamics but from global probability distribution-dynamics (see Materials and Methods), for which the traditional approaches fail. Our method transforms stochastic state-dynamics to deterministic distribution-dynamics, since one set of the variables of the high-order moment system at one time point corresponds to one probability distribution of state at that time point. Thus, the signals are actually detected from the high-order moment system with smaller noise rather than from the original system with big noise, therefore, we call the criterion as the moments-based DNM score. Theoretically, DNM, which is a model-free approach, can be applied to a wide class of systems as a generic indicator, regardless of differences in the details of respective systems, provided that the processes are accompanied with state or distribution transition phenomena.

To demonstrate the effectiveness and efficiency of DNM, we applied our method to a simulated dataset and three real datasets, i.e., the genomic data on lung injury induced by carbonyl chloride inhalation exposure (GSE2565)^31^, the ecological data on a critical transition to a eutrophic lake state^32^, and the financial data on the bankruptcy of Lehman Brothers^26^, for which we all successfully identified the critical or pre-transition states.

## Results

### Detecting early-warning signals of critical distribution-transition with big noise by “making big noise smaller”

**Figure 1:**
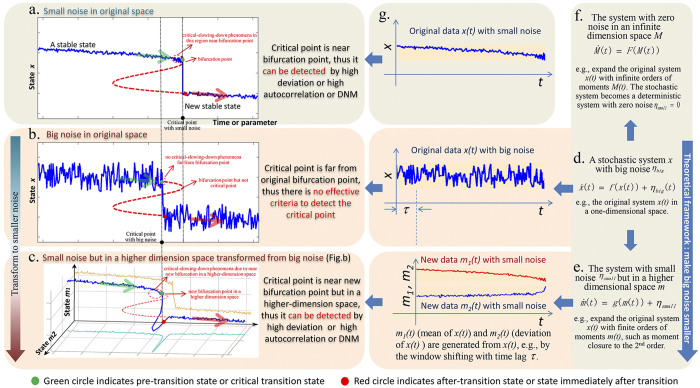
Scheme of probability distribution embedding. (**a,b**) show different types of dynamical behavior of a system with the gradual change of the parameter or time when it is under small noise and under big noise, respectively. (**a**) When the system is under small noise, the critical point of the system is near a bifurcation point of the corresponding deterministic system, in which there is a critical-slowing-down (CSD) phenomenon (e.g., a one-state-variable system). Thus, CSD can be used to detect its signals because signals of CSD only appear when the system approaches the bifurcation point. (**b**) When the system is under big noise, the critical transition takes place much earlier than that of the deterministic system due to strong fluctuations. There is no CSD phenomenon since the transition is far from the original bifurcation point. Thus, we cannot directly apply CSD to identify the critical point. (**c**) By moment expansion, the state-dynamics under big noise is transformed to the probability distribution-dynamics with much smaller noise but in a higher-dimensional space (e.g., a two-moment-variables system), for which the critical point is near the bifurcation of the reconstructed high-dimensional system. Thus CSD-based method works effectively again and can be used to detect early-warning signals in this higher-dimensional system. Note that we aim to identify the pre-transition state rather than the state after the critical transition. (**d**) shows the original dynamical system with one variable and the observed time-series data with big noise. (**e**) shows the expanded moment system with two variables from (**d**) and the reconstructed time-series data in a higher-dimensional space but with smaller noise. (**f**) shows an extreme case, for which the original system with big noise can be expanded to an infinite-dimensional system with zero noise. Generally, a linear stochastic system with big Gaussian noise can be exactly represented by up to the 2nd-order moment system with zero noise, i.e., Gaussian distribution. The dotted red lines are unstable equilibria, which separate the two basins of the two stable equilibria. We detect the green circle rather than the red circle.

Many real systems are fluctuated by big noise. Typical examples include ecosystems, biomolecular systems, and financial systems, whose dynamics are all convoluted with strong noise. With big noise, the critical point is actually far from the bifurcation point (Fig. 1b), i.e., the critical transition may occur stochastically far before the deterministic bifurcation point under the perturbations of big noise. Actually, we will show that this transition is not deterministic (or traditional) state-transition but stochastic distribution-transition. However, such earlier transition cannot be predicted by CSD since the strong nonlinearities around the transition point violates the assumption of the CSD for a linear restoring force, and thus those traditional criteria based on CSD are not suitable for the systems with big noise. Note that we do not consider the flickering phenomenon in this paper, i.e., we assume only to have the observed data before the transition to another state, and thus the transition is actually the conditional probability distribution transition.

We developed a theoretical framework, i.e., distribution embedding, to transform the observed original data (state-variables) with big noise into new synthetic data in a higher-dimensional space (distribution-variables) but with smaller noise (Fig. 1), i.e., by converting the observed state-dynamics into the distribution-dynamics (Methods, Fig. S4). In such a way, the criteria based on CSD works again on the new data in the high-dimensional space due to the smaller noise. Moreover, we detect the pre-transition state by DNM by analyzing the new data in the high-dimensional space (Table S1). Specifically, increasing the dimension of the observed data (representing the state) by moment expansion, we can derive new data (representing the probability distribution, i.e., conditional probability distribution) but with much smaller noise (Methods, Fig. S4), where a set of moments correspond a probability distribution. Thus, based on the transformed data by DNM, we can predict the distribution-transition, which results in the drastic change of the distribution, rather than the traditional state-transition. Note that we can only observe the data in the original state before the transition, and have no information on the state after the transition, i.e., no flickering. Thus, the observed probability distribution is the conditional distribution.

Generally, a dynamical system with big noise can be expressed by the following stochastic differential equation


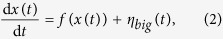


where 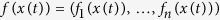 are nonlinear functions, state variables are 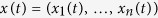, and noises are 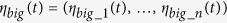 with mean 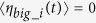 and covariance 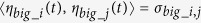. Here the angle brackets 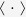 is the operator for calculating the average.

Then, we can approximate system (2) by the following moment evolution equation^33^ with moment expansion to the *k*th order:


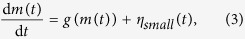


where 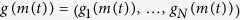 are nonlinear moment functions derived from 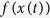, and moment variables are 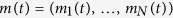. Due to truncation to the *k*th order of moments, the error functions are 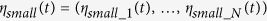, which can be taken as noise terms. 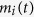 is a moment, and *N* is the total number of the moments up to the *k*th order. In particular, if expanding the moments to *k* = 2, then 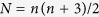, where the moment variables 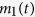 are means (the first order moment of variable 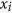, i.e., 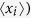 and 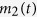 are covariances (the second order central moments of variables 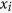 and 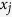, i.e., 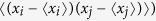 of 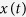. Actually, to approximate the original stochastic dynamics or minimize the error terms 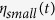 by finite order moment equations (3), many sophisticated schemes to truncate moments, such as moment closure^34^,^35^, have been proposed. By this moment-system with smaller noise, we can directly use DNM to detect the critical transition, where the critical point is not the bifurcation point of the original system (2) but the one of (3). Note that any probability distribution can be represented or expanded by Gram-Charlier, Edgeworth series^36^ or Binomial moment series^37^ in terms of moments 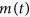. Hence, a set of moments represent one probability distribution, i.e., this moment-system represents the dynamics of the state probability-distribution rather than the state dynamics of the original system 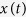, and thus the critical point of the moment-system (3) corresponds to the drastic change of the probability-distribution rather than the drastic change of the state 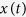. In other words, different from the critical state-transition of the deterministic system in terms of 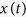, the transition of the stochastic system (3) in terms of 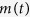 is the critical distribution-transition. We presented the detailed derivation of the distribution embedding as well as the algorithm in SI A.3.

To illustrate the effect of noise reduction by the distribution embedding, we employ an one-dimensional mathematical model; and then to demonstrate DNM, we effectively detect early-warning signals for three real-world problems by using observed data, i.e., a pre-disease state of lung injury, a critical point of a eutrophic lake state, and a catastrophic phenomenon of financial markets, which are all fluctuated with big noise.

### Identifying pre-transition states in small noise and big noise by DNM

Hereto we elucidate the distribution embedding approach by a simulated example, i.e., consider that data 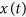 are observed from a dynamical system expressed by


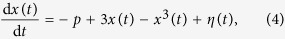


where 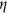 is a white noise with zero mean, i.e., the mean 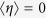 and the variance 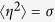. Note that in real situations, the model or system (4) is generally unknown to us.

**Figure 2:**
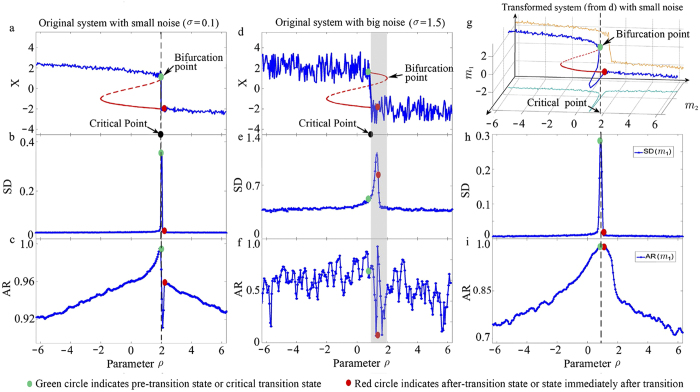
Traditional criteria based on CSD under small noise and big noise. (**a–c**) show the state-variable of Eq. (4) and statistical indices, i.e., the standard deviations (SD) and the autocorrelations (AR), respectively when the system is under small noise 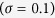. (**d–f**) show the state-variable of Eq. (4) and statistical indices, i.e., the standard deviations (SD) and the autocorrelations (AR), respectively when the system is under big noise 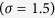. The green spot represents the pre-transition or critical state, while the red one is the state immediately after the transition. (**a**) The state-variable under 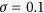. (**b**) The standard deviation under 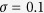. (**c**) The autocorrelation under 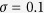. (**d**) The state-variable under 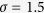. (**e**) The standard deviation under 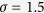. (**f**) The autocorrelation under 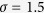. It can be seen from (**a–c**) that SD and AR can signal the emergence of the critical transition under a small noise. However, (**d–f**) show that SD and AR cannot reflect the critical transition under big noise. (**g–i**) show the moment-variables of Eqs (5,6) and statistical indices, i.e., the standard deviations (SD) and the autocorrelations (AR), after increasing the dimension from 1 to 2. (**g**) the moment-variables under 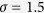 derived from (**d,h**) the standard deviation of 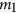 under 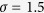. (**i**) The autocorrelations of 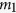 under 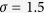. It can be seen that the CSD-based criteria work again.

The system (4) has a bifurcation point around 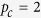 (see Fig. 2a and Fig. S5 of SI) when ignoring the noise 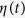. We generate time-course data from (4) by changing parameter *p* from −6 (one stable state) to 6 (another stable state) with small noise 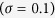 shown in Fig. 2a. With small noise 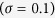, the critical slowing down (CSD) phenomenon^4^ appears when the system approaches the bifurcation point, and thus the traditional statistical indices based on the CSD principle, i.e., standard deviation (SD), covariance (COV), autocorrelation (AR) and skewness are able to signal the emergence of the state-transition as the parameter *p* approaches the bifurcation value 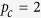 (Fig. 2b,c). However, when the system is under big noise 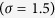, the critical transition appears much earlier than the bifurcation point, due to the strong perturbations. Thus those indices based on the original state-dynamics fail to indicate the earlier transition. Actually, we generate the time-course data from (4) with big noise 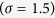 shown in Fig. 2d, which demonstrates that the regime shift of the system occurs around 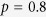 far earlier than 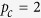 (Fig. S8). In this case, the CSD principle and other methods based on the eigenvalue fail to detect the pre-transition state (Fig. 2e,f).

For the purpose of illustration, next we approximate (4) by the moment expansion up to the second order (see SI B for details), although our method is only based on the observed data and does not require this analytical implementation:


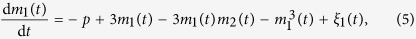



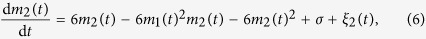


where 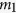 is the mean (the 1st-order moment), 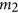 is the variance (the 2nd-order central moment), and *σ* is the amplitude or variance of original white noise 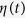. 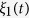 and 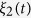 are considered as small noises derived from moment closure. Clearly, 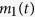 and 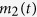 approximately represent the distribution of the original state 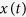 in (4). For system (5)–(6) without the noise terms, there is a bifurcation point around 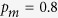 much earlier than the original 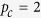 (see Fig. S5 and Fig. S8 in SI), which implies the critical distribution-transition is expected around 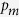 (see Fig. S6 and Fig. S7), and thus quite different from the traditional state-transition.

Based on the data 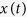 in Fig. 2d and the computational procedure with window interval 10 (see SI A.3 and A.5), we construct the synthetic time-course data of 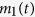 and 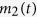 (Fig. 2g), where the noise level is clearly smaller than that of the original in Fig. 2d. Actually, the standard deviations of 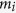 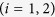 are less than 0.4 while that of original variable *x* is more than 1 according to the simulation results. Obviously, the critical point is near the bifurcation point when the system is under small noise (Fig. 2a), while the critical point is far ahead of the bifurcation point when the system is perturbed by big noise (Fig. 2d). It should be noted that the bifurcation point of 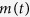 is moved to around 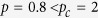 (Fig. S8). Then, instead of the original system *x* with big noise, we study the moment system 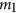 and 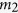 with small noise. Thus, for this moment system, SD and AR are again sensitive to the critical transition (Fig. 2h,i) due to the small noise level. It can be seen that the critical point of the original stochastic system is again close to the bifurcation point of the transformed system (Fig. 2g). Notice that the two new state-variables behave in a strongly correlated manner in dynamics when the system approaches the transition point. Hence the DNM score suffices to signal the critical transition before its occurrence. Note that Fig. S8 also shows that the critical distribution-transition point of the original system is approximately the bifurcation point of the moment system for both big noise 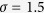 and small noise 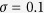.

Different from the traditional (critical) “state-transition” for a deterministic system, the critical transition caused by big noise is actually a critical “distribution-transition” for a stochastic system, that is, the distribution of the state for the stochastic system has a drastic change from one to another, which results in a new probability distribution (Fig. S12). By such a transition, the probability of the current stable state can be significantly reduced while the probability of another stable state may be drastically increased. The distribution of such a critical distribution-transition is related to the magnitude of noise, that is, the larger the noise is, the earlier the critical distribution-transition would be; the nearer to the bifurcation point, the more probable that the system would transit into a new stable state. We give a detailed discussion in SI B and Fig. S1. Note that we do not consider the flickering phenomenon in this paper, i.e., we assume only to have the observed data near the original stable state before the critical transition to another state, and based on such observed data, to detect the early-warning signals of the critical transition.

### Predicting critical transition in a network

**Figure 3:**
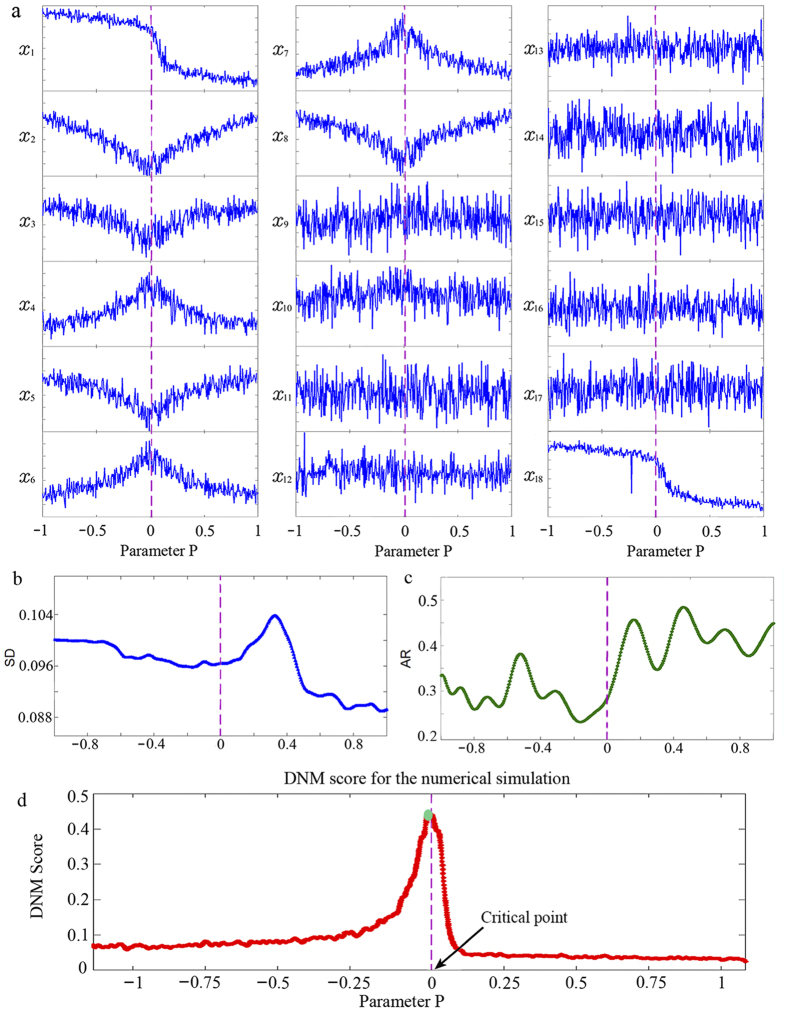
Predicting critical transition in a 18-node network by DNM. (**a**) shows dynamics of the 18 nodes by slowly changing the value of the parameter *P*. It is difficult to indicate the critical transition of this trajectory from single variables (or their SDs and ARs) since the strong fluctuations of variables appear at almost all of variables due to the big noise. (**b,c**) respectively show the CSD-based indices, i.e., SD and AR, which fail to signal imminent critical transition at *P* = 0. (**d**) The curve shows the clear increasing tendency of the DNM score before the critical point at *P* = 0, and thus the DNM score suffices to signal the critical transition before it occurs. The pre-transition state of this trajectory is presented as the green circle.

We employ an eighteen-node gene regulatory network (shown in Fig. S9) to demonstrate the effectiveness of DNM. The detailed descriptions of the network represented by a set of stochastic differential equations are provided in SI C, and numerical simulations are provided in Fig. 3. It is difficult to use traditional SD or AR to detect the signals from *x* due to multi-variables and big noise. Also, the change of the largest singular value cannot signal the imminent critical transition due to noisy data and a small number of samples (see Fig. S10). We transformed the data into the moment-variable (or equivalent distribution) data by *k* = 2, and the total moment variables are 189 with window interval 10. In contrast, our computation result shows that a drastic change (or sharp increase) in the DNM (27 variables) score indicates the emergence of a critical state of this trajectory just before the critical transition, which validates that the DNM can serve as a general indicator by detecting the early-warning signal of the system.

### Predicting critical transitions in real datasets

**Figure 4:**
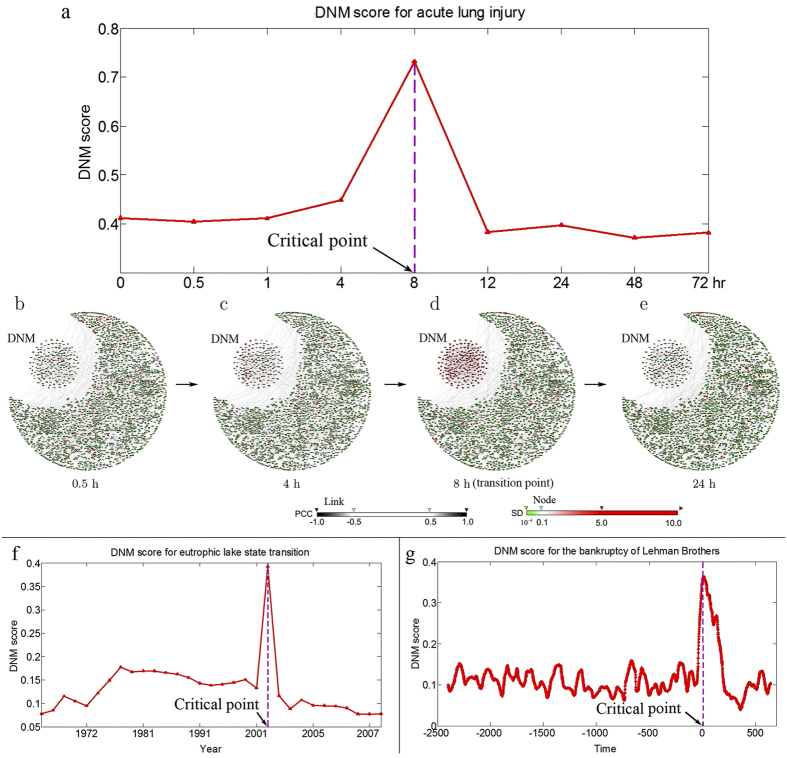
The applications of DNM on three real data. (**a**) The DNM score for acute lung injury clearly shows an signal around 8 hr, that is, an abrupt increasing of DNM from 4–8 hr, around which a critical transition occurs. (**b–e**) The figures show the dynamical changes of the molecular network at (**b**) 0.5 hr, (**c**) 4 hr, (**d**) 8 hr, and (**e**) 24 hr with the corresponding DNM, where the color of nodes represents the fluctuation strength in gene expressions, and each edge represents the correlations between two nodes. A small cluster located in the upper-left corner is the identified DNM-related genes. It can be seen that at 8 hr, there is a strong signal to indicate the pre-transition state. (**f**) The DNM score for a eutrophic lake state clearly shows a signal around year 2002, at which a critical transition occurs. Plots were calculated by employing a 59-year (half time series) sliding-window during the period 1883–2009, and are plotted to the right of the window. (**g**) The DNM score for the bankruptcy of Lehman Brothers clearly shows an signal around the time point 0 (2008/9/15), at which a critical transition occurs. Plots were calculated by employing a 100-trading-days sliding-window, and are plotted to the right of the window.

We further applied DNM to three real datasets, i.e., the microarray data for acute lung injury induced by carbonyl chloride inhalation exposure (GSE2565) which records the time-course microarrays collected from lung tissue of mouse^31^, the ecological dataset about the eutrophic lake state which records the historical yearly data for lake-water-quality indices^32^, and the financial dataset about the bankruptcy of Lehman Brothers which records the historical daily prices of interest-rate swaps in the USD and EUR currency^26^. The detailed computation and data description are described in SI D and E, respectively. Figure 4 shows the identified pre-transition states just before the critical deteriorations by the DNM score, all of which well agreed with the observed transition phenomena described in the original datasets^26^,^31^,^32^.

Figure 4a presents the DNM (169 variables) scores for acute lung injury (total 12,871 observed variables or genes in the original state space), which shows that there is a clear signal, that is, the DNM score increases sharply and peaks at 8 hr. Therefore, we identified the pre-transition state around 8 hr. In the original experiment, a 50%–60% mortality was observed at 12 hr and a 60–70% mortality was observed at 24 hr^31^, which agrees with our result. It can be seen from Fig. 4a that the DNM score indicates the signals of the pre-transition state before the critical point. Further, a figure illustrating the dynamical changes of the whole molecular network from 0.5 hr to 24 hr is shown in Fig. 4b–e, where a strong signal for the pre-transition state can be observed around 8 hr (also see Fig. S11 for the whole progression of the disease). Therefore, the DNM score is able to identify the pre-transition state, which is consistent with our previous results^2^,^28^ and the observed experimental results^31^. The identified DNM variables are listed in the Supplementary Table ‘Identified DNM members A’.

Figure 4f shows the change of the DNM (21 variables) score for the eutrophic lake state (total 11 observed variables in the original state space, and total 77 moment variables in the moment expansion space), which is constructed from recorded data of historical changes in the Erhai Lake catchment system in Yunnan, China^8^. It can be seen that the DNM curve peaks near the critical transition of the eutrophic lake state (around year 2002) and thus presents a clear signal for the critical state-transition, which well agrees with the ecological records, i.e., the original records show an abrupt transition in algal states between 2001 and 2005. From the combined monitored and lake sediment data, it seems that a profound transition in the algal community occurred around 2002^32^. It is also pointed out that the transition in Erhai Lake in 2002 corresponds to the classic development of a bistable system^32^, that is, the shift in the state of the diatom communities and the abrupt changes in water quality indicators are consistent with the behaviour of the lake that is shifting from a stable state (i.e., the oligotrophic state) to another stable state (the eutrophic state). Therefore, DNM correctly predicted the imminent transition from one state to another. The identified DNM variables are presented in the Supplementary Table ‘Identified DNM members B’.

The critical transitions in financial market are often referred to the broken of unstable “financial bubbles”. For the data of financial market related to the bankruptcy of Lehman Brothers, which was once the fourth-largest investment bank in the United States before declaring bankruptcy on September 15, 2008 and whose bankruptcy is thought to have played a major role in the unfolding of the late-2000s global financial crisis, the traditional criteria based on CSD (i.e., SD and AR) failed to signal the occurrence of critical transition (see Fig. S11 in SI) possibly due to strong fluctuations of data. However, as shown in Fig. 4g by using our scheme, the DNM (5 variables) score increases abruptly before the bankruptcy (total 5 observed variables) of Lehman Brothers (time point 0), which is consistent with the phenomena^26^, and this result clearly shows the effectiveness of DNM to apply to financial collapse prediction.

The successful applications of DNM in the three real datasets show the effectiveness of DNM in identifying the pre-transition states even with big noise or perturbation. The detailed computational procedure and data description are provided in SI D and E, respectively. The identified DNM for the biological dataset is also given in Supplementary Table ‘Identified DNM members’. To validate the effectiveness of the noise reduction by our method, we also conducted the calculation of the signal-to-noise ratio (SNR) for the three real datasets shown in Table S2 (Supplementary Information), and the results indicated that SNRs in the high-dimensional space were all increased after the implementation of the moment expansions, compared with those in the original space.

## Discussion

To detect early-warning signals of critical transitions for complex dynamical systems with big noise, first we developed a distribution embedding scheme, by increasing the dimension of the original data, and secondly we extended CSD (for single variables) to DNM (for multi-variables or a network) so as to obtain robust signals at a network level by exploring correlation and fluctuation information of high-dimensional data.

In this work, we raised a concept, i.e., distribution-transition, in contrast to the traditional state-transition. A state-transition occurs as deterministic bifurcation of a system with small noise, which can be detected by the traditional method CSD or DNM. However, when the system is perturbed by big noise, the transition occurs far earlier than the deterministic bifurcation point of the original system, which makes the traditional methods fail. In this work, we show that such a system can be transformed into a moment-system with small noise. Thus, traditional method CSD or DNM can be used to detect the critical point or bifurcation point of the moment system. Since a set of moments of 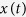 correspond to one probability distribution of 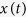, the bifurcation of the moment system with finite terms of moments also approximately corresponds to the critical point of the probability distribution. Therefore, such a transition is the distribution-transition, which results in the drastic change of the distribution.

To overcome the problem of big noise, the key idea is to change the observed state-dynamics with big noise to the probability distribution-dynamics with much smaller noise, which can be represented by moment dynamics. Thus, due to the reduced fluctuation or noise level on the distribution-dynamics, the traditional indices or methods based on CSD can be directly applied to the data of the transformed distribution-dynamics rather than the data of the original state-dynamics. Such a transformation from the state to the distribution clearly increases the dimension of the system. As indicated in the results, our method can exploit the information of both fluctuations and correlations between observed variables in a multi-dimensional system, in contrast to the CSD-based criteria which mainly focus on the fluctuations of individual variables. It should be noted that the distribution-dynamics represented by moments does not increase the amount of information but just reduces the level of the noise because some part of noise is embedded into the distribution or deterministic model, so that we can accurately detect the early-warning signals for the transformed system with small noise. As shown in Fig., the bifurcation point of the moment system corresponding to the distribution-transition indeed moves earlier with the increase of the noise level. From the viewpoints of both theoretical analysis and numerical computation, we demonstrated that DNM is sensitive to the pre-transition state and suffices to provide early-warning signals for the critical transition even if the related dynamical model is unknown and the original data are not reliable due to the big noise. Note that we can only observe the data around the present stable state before the transition to another state, and thus the transition is actually a conditional distribution transition due to no information available on another state.

Our method is able to identify the pre-transition state before the critical distribution-transition, rather than the after-transition state, and therefore has great potential to apply to many real systems even with strong noise. It is also worth noting that the members in DNM make the first move from the before-transition state toward the after-transition state during a transition, and thus may be causally related with transition-driving factors. Hence, those members in DNM have significant physical or biological implications depending the subjects under study. Although a major advantage of increasing dimensionality is the reduction of the noise level, it requires additional data to construct the time-series of higher order moments. It is a future topic how to construct the higher-dimensional system with short time-series data by efficiently exploring information of correlations and dynamics among the observed variables^38^,^39^. Also it should be noticed that although there are many ways for moment expansions, such as Gram-Charlier or Edgeworth series, which are not convergent series, it is of importance to find an appropriate expansion scheme to accurately reconstruct the system with higher dimensions.

## Methods

### Theoretical basis to detect early-warning signals of critical distribution-transition with big noise by distribution embedding

When the system is fluctuated by big noise, the critical point is far earlier than the bifurcation point, which may make the critical slowing-down principle fail. However, we can transform the stochastic system into moment equations, a set of ordinary differential equations (ODEs) with moments as variables, that is, the mean, variance, skewness and so on, and thus reduce the level of the original noise. A set of moments correspond to a probability distribution, and such a transformation is actually to convert the state dynamics into the distribution dynamics. In the following, we explain such a procedure based on dynamical systems theory.

For a linear system, Eq. (2) can even be exactly expressed by Eq. (3) with the moment expansion up to the second order, i.e., *k* = 2. For this case, there is no error, i.e., the noise is reduced to zero, 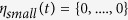. For a nonlinear system, if *x* follows Gaussian distribution, Eq. (2) can also be exactly expressed by Eq. (3) with *k* = 2 and the zero error.

For a general nonlinear stochastic system, with moment expansion to an infinite order^33^, i.e., as 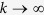, the dynamics of system (2) can be expressed by Eq. (7) in an exact manner, which becomes a deterministic system with the zero error or noise.


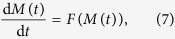


where 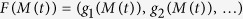 are nonlinear moment functions, and moment variables are 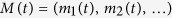. The error functions are reduced to zero, i.e., 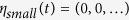. Also see the intuitive explanation in Fig. 1.

In other words, it is expected that, the higher the order of moment expansion is, the more accurately the resulting dynamics (3) would approximate that of the original system (2) in terms of the distribution, and thus the smaller the noises or error terms are^37^. This result gives the theoretical basis to reduce the noise level by increasing the dimension of the original system. In particular, the moment system corresponds to the distribution dynamics, i.e., a set of moments represent one distribution. Thus, Eq. (3) or (7) can be also viewed as the transformation from state dynamics with big noise to distribution dynamics with small noise. Note that we can only observe the data in the original state before the transition, and have no information on the state after the transition, i.e., the state is assumed to have no flickering. Thus, the observed probability distribution is the conditional distribution. Also note that in real situations, our analysis is only based on the observed data, and does not need the above analytical implementations. Next we will describe the implication of the critical transition for the moment-system, and then give the detail procedure to construct the synthetic data in a higher-dimensional space from the observed original data.

We specifically derive the moment evolution equations by expanding the moments to the second order, i.e., *k* = 2. Let the first-order moment (or mean) be 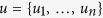 with 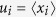, and the second-order moment (or covariance) be 
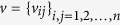
 with 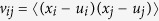. Then the moment equations are given by the following deterministic system^33^:


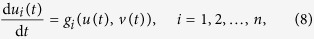



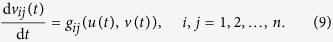


where


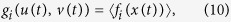



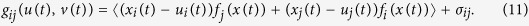


Therefore, the original stochastic system (2) is transformed to a deterministic system (8)–(9).

If the original system is linear, that is, 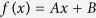, where 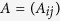 is an *n* × *n* constant matrix and 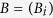 is a constant *n*-dimensional vector, then obviously we can analytically derive the moment system (8)–(9) directly, due to


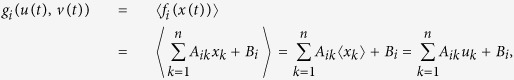



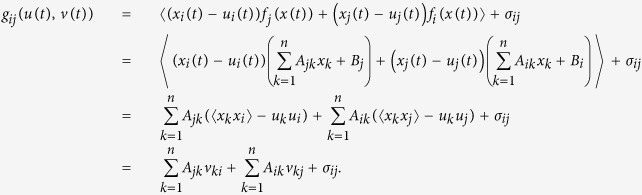


Thus, the original system can be analytically expressed by the first-order moments *u* and the second-order moments *v*.

However, if the original system is nonlinear, the deterministic system (8)-(9) is generally unclosed with the first and second order moments. That is, in the expressions (10) and (11) there are usually involved with high-order moments, namely the third or higher order moments. To circumvent this problem, the approximation methods, such as moment-closure^40^, are used to truncate moments up to the second order, thereby making Eqs. (8)-(9) closed in terms of the first and second order moments. Due to such an approximation, there are additional error or noise terms in 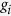 and 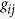 of Eqs. (8)-(9), as described in Eq. (3). Note that we can make similar analysis by using binomial moments.

### Data processing

The gene expression profiling dataset for lung injury disease was downloaded from the NCBI GEO database (ID: GSE2565) (www.ncbi.nlm.nih.gov/geo). The networks were visualized using Cytoscape (www.cytoscape.org). The detailed description and data processing were presented in SI E1.

For the ecological dateset of a eutrophic lake state, the data were sampled during the period 1883–2009^32^, including historical trends for lake water quality, and several related chemical indices. We used the sliding-window method with a 59-year period. The detailed background of this dataset can be found in SI E2.

The financial dataset was from the ING Bank and consists of the time series of USD and EUR interest-rate swaps (IRS)^26^. The data span more than twelve years: the EUR data from 12/01/1998 to 12/08/2011 and the USD data from 04/29/1999 to 06/06/2011. Here, we only used the mean of the daily prices of IRSs in the USD and EUR currency. The introduction of this dataset was in SI E3.

## Additional Information

**How to cite this article**: Liu, R. *et al*. Identifying early-warning signals of critical transitions with strong noise by dynamical network markers. *Sci. Rep*. **5**, 17501; doi: 10.1038/srep17501 (2015).

## Supplementary Material

Supplementary Information
