# Supplementary material for: Identifying early-warning signals of critical transitions with strong noise by dynamical network markers
Source: Sci Rep. 2015 Dec 9;5:17501. doi: 10.1038/srep17501 (PMC4673532; doi:10.1038/srep17501)
Supplement: Supplementary Information [file srep17501-s1.pdf]

Supplementary Information: Identifying early-warning signals of critical transitions with strong noise by dynamical network markers

Rui Liu, Pei Chen, Kazuyuki Aihara, Luonan Chen

Contents

A Theoretical background **S3**

A.1 Dynamical network marker near critical transition point with small noise S3

A.1.1 Dynamical network marker near saddle-node or period-doubling bifurcation point with small noise S5

A.1.2 Dynamical network marker near Neimark-Sacker bifurcation point with small noise S10

A.1.3 Dynamical network marker for nonlinear systems near critical transition point with small noise S18

A.2 Three states during a critical transition process S21

A.3 Distribution embedding near critical transition point with big noise by moment expansion S22

A.4 Critical state-transition and critical distribution-transition S28

A.5 Constructing time-course data of moments in a higher-dimensional space from original data S29

B One-dimensional example **S30**

B.1 Moment-system S30

B.2 Critical state-transition represented by states, and critical distribution-transition by moments S32

B.3 Bifurcation point of the moment system corresponding to the critical distribution-transition moves earlier with the increase of noise level S34

C Numerical validation of DNM **S36**

C.1 Validation of DNM S36

C.2 Comparison with SVD S38

D Algorithm for calculating DNM **S39**

E Application to three real datasets **S41**

E.1 Dataset 1. Genomic data of the lung injury with carbonyl chloride inhalation exposure (i.e., acute lung injury) S42

E.2 Dataset 2. Ecological data about the eutrophic lake state S46

E.3 Dataset 3. Financial data about the bankruptcy of Lehman Brothers S49

F State-transition with small noise and distribution-transition with big noise **S51**

G Comparison of distribution embedding scheme with support vector machine **S51**

H The signal-to-noise ratio of the three datasets **S54**

A Theoretical background

A.1 Dynamical network marker near critical transition point with small noise

For a complex dynamical system with multiple variables or network, assume that we measure the variables at different time points. In this section, we theoretically introduce several generic properties of such a dynamical network when the system approaches a critical transition point. Specifically, we derive the conditions to obtain dynamical network marker (DNM) in a general system or dynamical network biomaker (DNB) in a biological system, which can characterize the generic properties and predict the critical transition, based on bifurcation theory and center manifold theory (*6, 7*).

We consider the following discrete-time dynamical system that represents the dynamical evolution of a network:

$$Z(t+1) = f(Z(t); P), \tag{S1}$$

where $Z(t) = (z_1(t), ..., z_n(t))$ is an n-dimensional state vector or variables at time instant t, while $P = (p_1, ..., p_s)$ is a parameter vector or driving factors that represent slowly changing factors. $f : \mathbf{R}^n \times \mathbf{R}^s \to \mathbf{R}^n$ are generally nonlinear functions.

Furthermore, we assume that the following conditions hold for Eq.(S1).

1. $\bar{Z}$ is a fixed point in system (S1) such that $\bar{Z} = f(\bar{Z}; P)$.
2. There is a value P_c such that one or a pair of the eigenvalues of the Jacobian matrix $\frac{\partial f(Z;P_c)}{\partial Z}\Big|_{Z=\bar{Z}}$ is equal to 1 in the modulus.
3. When $P \neq P_c$, the eigenvalues of (S1) are not always equal to 1 in the modulus.

These three assumptions with other transverse conditions (*1*) imply that the system undergoes a phase change at $\bar{Z}$ or a codimension-one bifurcation when P reaches the threshold P_c.

From a mathematical perspective, the bifurcation is generic, *i.e.* almost all of the bifurcations in a general system satisfy these conditions. It is notable that most of the systems described by differential equations can be generally discretized and transformed into Eq.(S1), e.g., using methods such as the Euler scheme and the Poincaré section. Thus, we focus on difference equations (S1) during our theoretical analysis in this section.

It is known that the dynamics of a nonlinear system is highly complex far before or after a sudden transition; therefore, the state equations of systems are generally constructed in a very high-dimensional space using a large number of variables and parameters (*1–4, 15, 17*). However, if a system driven by known or unknown parameters approaches a critical point, which is a very special phase during its dynamical evolution, it is theoretically guaranteed that the system will eventually be constrained to one- or two-dimensional space (i.e., the center manifold), which can be expressed in a simple form around a codimension-one bifurcation point (*5,6,15,17*). This is generally guaranteed by the bifurcation theory and the center manifold theory (*5–8*). Thus, we can detect the signal of any dynamical system only during this special phase and not during other periods (i.e., neither the before-transition state nor the after-transition state), which is part of the theoretical foundation of this study (*15, 17*).

For system (S1) near $\bar{Z}$ and before P reaches P_c, we assume that the system is at a stable fixed point $\bar{Z}$, so all of the eigenvalues are within $(0, 1)$ in the modulus. The parameter value P_c when the state shift of the system occurs, is known as a bifurcation parameter value or a critical transition value.

This theoretical result was derived based on consideration of the linearized system or equations for Eq.(S1) and the small noise perturbations near $\bar{Z}$. Specifically, by introducing the new variables $Y(t) = (y_1(t), ..., y_n(t))$ and a transformation matrix S, *i.e.*

$$Y(t) = S^{-1}(Z(t) - \bar{Z}),$$

we have

$$Y(t+1) = \Lambda Y(t) + \zeta(t). \quad \text{(S2)}$$

where $\Lambda(P)$ is the diagonalized matrix of $\frac{\partial f(Z;P)}{\partial Z}\Big|_{Z=\bar{Z}}$. $\zeta(t) = (\zeta_1(t), ..., \zeta_n(t))$ are small Gaussian noises with zero means. We denote σ_i as the small standard deviation of ζ_i for all k. Without any loss of generality, the diagonalized matrix $\Lambda(P) = diag(\lambda_1(P), ..., \lambda_n(P))$ for each $|\lambda_i|$ is between 0 and 1. In view of the dominant eigenvalue (the largest eigenvalue in modulus), there are two typical cases during the diagonalization process (*15*), i.e., the dominant eigenvalue is real (including multiple real dominant eigenvalues), and the dominant eigenvalues are a pair of complex conjugate values. When the modulus of the largest eigenvalue or eigenvalue pairs approaches 1, there are three generic codimension-one bifurcations of the system, corresponding to these cases.

A.1.1 Dynamical network marker near saddle-node or period-doubling bifurcation point with small noise

We first illustrate the case of real dominant eigenvalue. Without loss of generality, actually, it is the diagonal case with real eigenvalues. For such a case, the critical point is the saddle-node bifurcation if the largest eigenvalue approaches 1, while the critical point is the period-doubling bifurcation if the largest eigenvalue approaches -1.

Among the eigenvalues of Λ, the largest one (in the modulus), say λ_1, approaches 1 in the modulus when parameter $P \to P_c$. It should be noted that there may be more than one real dominant eigenvalues (the case of multiple roots), for which the derivation is similar (*15*). Thus we just discuss the case with a unique real dominant eigenvalue λ_1, i.e., a generic case. The eigenvalue λ_1 characterizes the system's rate of change around a fixed point and is known as the dominant eigenvalue. The before-transition state corresponds to a period where $|\lambda_1| < 1$, whereas the pre-transition stage corresponds to the period with $|\lambda_1| \to 1$. Without loss of

generality, we assume that the first variable y_1 in Y corresponds to λ_1, namely $(y_1, 0, ..., 0)$ is the eigenvector of λ_1.

Because Λ is a fully diagonalized matrix, we have the variance

$$\mathrm{Var}(y_i) = \frac{\kappa_{ii}}{1-\lambda_i^2},$$

and the covariance

$$\mathrm{Cov}(y_i, y_j) = \frac{\kappa_{ij}}{1-\lambda_i\lambda_j}.$$

See (*15, 17*) for the detailed derivation. Hence when $|\lambda_1| \to 1$, $\mathrm{Var}(y_1) \to +\infty$. Note that $0 \leq |\lambda_1| < 1$ before the critical transition. Other λ_i $(i = 2, 3, ..., n)$ satisfy $0 \leq |\lambda_i| < 1$, and $\mathrm{Var}(y_i)$ approaches some positive bounded value.

With respect to the original state space, noting $z_i(k) = s_{i1}y_1(k) + \cdots + s_{in}y_n(k) + \bar{z}_i$, the variance is

$$\begin{aligned}\mathrm{Var}(z_i) &= s_{i1}^2\mathrm{Var}(y_1) + \sum_{k=2}^{n} s_{ik}^2\mathrm{Var}(y_k) + \sum_{k,m=1,k\neq m}^{n} s_{ik}s_{im}\mathrm{Cov}(y_k, y_m) \\ &= s_{i1}^2\frac{\kappa_{11}}{1-\lambda_1^2} + \sum_{k=2}^{n} s_{ik}^2\frac{\kappa_{kk}}{1-\lambda_k^2} + \sum_{k,m=1,k\neq m}^{n} s_{ik}s_{im}\frac{\kappa_{km}}{1-\lambda_k\lambda_m}.\end{aligned}$$

When $s_{i1} \neq 0$, $\lim_{|\lambda_1|\to 1} \mathrm{Var}(z_i) \to +\infty$.

When $s_{i1} = 0$, $\lim_{|\lambda_1|\to 1} \mathrm{Var}(z_i) \to V_i$, where V_i is a bounded positive value.

As for the covariance and the correlation, we have

$$\begin{aligned}&\mathrm{Cov}(z_i, z_j) \\ &= \mathrm{E}((s_{i1}y_1 + \cdots + s_{in}y_n)(s_{j1}y_1 + \cdots + s_{jn}y_n)) \\ &= s_{i1}s_{j1}\mathrm{Var}(y_1) + \cdots + s_{in}s_{jn}\mathrm{Var}(y_n) + \sum_{k,m=1,k\neq m}^{n} s_{ik}s_{jm}\mathrm{Cov}(y_k, y_m) \\ &= s_{i1}s_{j1}\frac{\kappa_{11}}{1-\lambda_1^2} + \sum_{k=2}^{n} s_{ik}s_{jk}\frac{\kappa_{kk}}{1-\lambda_k^2} + \sum_{k,m=1,k\neq m}^{n} s_{ik}s_{jm}\frac{\kappa_{km}}{1-\lambda_k\lambda_m}\end{aligned}$$

When $s_{i1} \neq 0$ and $s_{j1} \neq 0$, $\lim\limits_{|\lambda_1| \to 1} \text{Cov}(z_i, z_j) \to +\infty$.

When $s_{i1} = 0$ or $s_{j1} = 0$, $\lim\limits_{|\lambda_1| \to 1} \text{Cov}(z_i, z_j) \to C_{ij}$, where C_i is a bounded value.

For Pearson's correlation coefficient (PCC)

$$\text{PCC}(z_i, z_j) = \frac{\text{Cov}(z_i, z_j)}{\sqrt{\text{Var}(z_i)\text{Var}(z_j)}}$$

$$= \frac{s_{i1}s_{j1}\frac{\kappa_{11}}{1-\lambda_1^2} + \sum\limits_{k=2}^{n} s_{ik}s_{jk}\frac{\kappa_{kk}}{1-\lambda_k^2} + \sum\limits_{k,m=1,k\neq m}^{n} s_{ik}s_{jm}\frac{\kappa_{km}}{1-\lambda_k\lambda_m}}{\sqrt{\left(\frac{s_{i1}^2\kappa_{11}}{1-\lambda_1^2} + \sum\limits_{k=2}^{n}\frac{s_{ik}^2\kappa_{kk}}{1-\lambda_k^2} + \sum\limits_{k,m=1,k\neq m}^{n}\frac{s_{ik}s_{im}\kappa_{km}}{1-\lambda_k\lambda_m}\right)\left(\frac{s_{j1}^2\kappa_{11}}{1-\lambda_1^2} + \sum\limits_{k=2}^{n}\frac{s_{jk}^2\kappa_{kk}}{1-\lambda_k^2} + \sum\limits_{k,m=1,k\neq m}^{n}\frac{s_{jk}s_{jm}\kappa_{km}}{1-\lambda_k\lambda_m}\right)}}.$$

When $s_{i1} \neq 0$ and $s_{j1} = 0$, $\lim\limits_{|\lambda_1| \to 1} \text{PCC}(z_i, z_j) \to 0$.

When $s_{i1} = 0$ and $s_{j1} = 0$, $\lim\limits_{|\lambda_1| \to 1} \text{PCC}(z_i, z_j) \to P_{ij}$, where P_{ij} is a bounded value.

Hence, close to a fixed point, among the original variables $Z = (z_1, ..., z_n)$ there is a dominant group or a DNM which is composed of variables $z_i = s_{i1}y_1(k) + \cdots$ with $s_{i1} \neq 0$. In other words, each DNM member is directly related to y_1. Therefore, for the case of a real dominant eigenvalue, we can prove that there is only one dominant group (i.e., DNM) (see Fig.S1). For this dominant group, we conclude that there are the following generic properties when the system approaches a critical transition point or tipping point (*15*).

Theorem 1 *We consider a stochastically perturbed linearized system for Eq.(S1). When P approaches the saddle-node or period-doubling bifurcation point, there is a dominant group, and the following results hold (also see Fig.S1).*

- *If both z_i and z_j are in the dominant group, then*

$$|\text{PCC}(z_i, z_j)| \to 1,$$

while $\text{SD}(z_i) \to \infty$ *and* $\text{SD}(z_j) \to \infty$;

- *if z_i is in the dominant group but z_j is not, then*

$$\mathrm{PCC}(z_i, z_j) \to 0,$$

  while $\mathrm{SD}(z_i) \to \infty$*, and* $\mathrm{SD}(z_j)$ *approaches a bounded value;*

- *if neither z_i nor z_j is in the dominant group, then* $\mathrm{PCC}(z_i, z_j)$ *approaches a constant, while both* $\mathrm{SD}(z_i)$ *and* $\mathrm{SD}(z_j)$ *approach bounded values,*

where PCC is the Pearson's correlation coefficient and SD is the standard deviation.

This dominant group of variables or elements is DNM. This theorem is the part of the theoretical basis of detecting the pre-transition state for multi-variable systems with small noise. The three conditions in the theorem are actually the criteria to detect the DNM (*15*). From the first criterion, we know that in a DNM group, each variable is strongly fluctuated due to the DNM nature in dynamics, and each pair of two variables in DNM are strongly correlated from the second criterion. In other words, DNM is a group of variables with strongly collective fluctuations, which indicates the emergence of the critical transition.

Actually, for saddle-node or period-doubling bifurcation, we have proved that there is only one dominant group (i.e., DNM) in the SI of (*15*) (see Fig.S1). Note that the theorem holds for a linearized system. The DNM is the subnetwork that makes the first move from one state toward the other at the critical transition point; therefore, we refer to the DNM as the leading network in this critical transition (*17*). For a general discrete-time dynamical system, all codimension-one bifurcations are transition points, including saddle-node bifurcation (transcritical and pitchfork bifurcation) if the dominant eigenvalue is equal to 1; period-doubling (or flip) bifurcation if the dominant eigenvalue is equal to -1; and Neimark-Sacker bifurcation if there is a pair of complex conjugate eigenvalues with modulus 1.

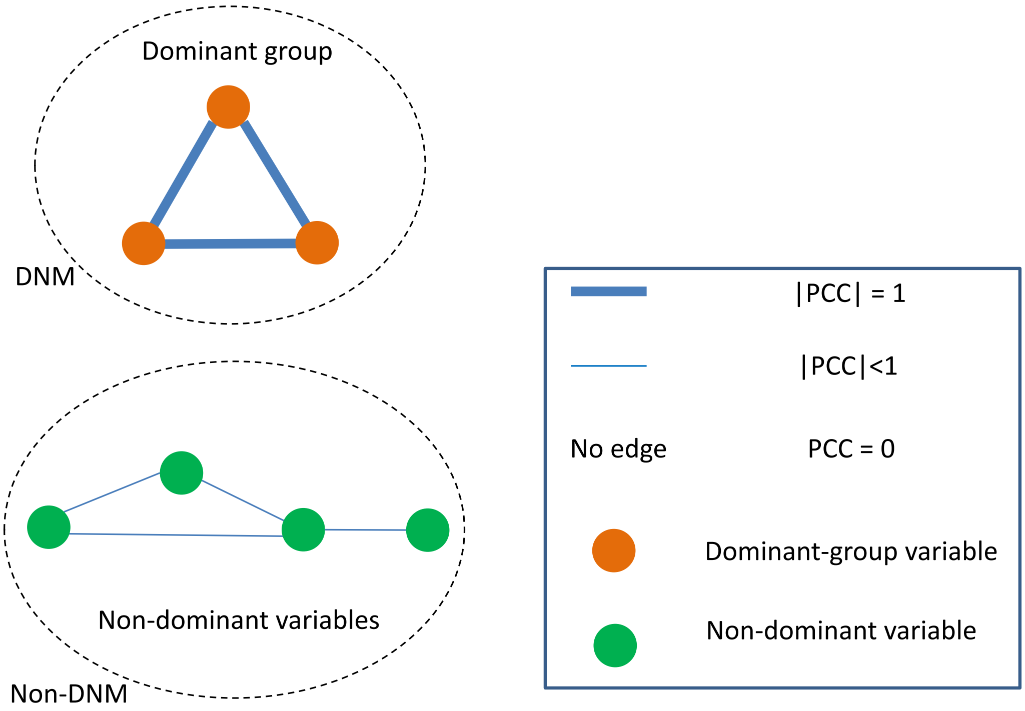

Figure S1: | **A sketch for the case of one dominant group (one DNM) just before the critical transition.** Under the assumption that the dominant eigenvalue is real, the sketch shows a network with one dominant group when the system approaches the critical point, where the orange nodes represent variables inside the dominant groups, while green nodes are outside. Each edge represents the correlation between two variables. It can be seen that among orange nodes in the dominant group, the correlations are strong. There is no correlation between a dominant-group member and a non-dominant-group node. Note that, if the system is not near the critical point, generally there are edges between nodes of the dominant variables and non-dominant variables, which are all in one network.

Note that there is only one DNM for any co-dimension-one bifurcation, except Neimark-Sacker bifurcation which corresponds to two DNMs with one quasi-DNM. For these two DNMs, they are all with strong SDs, but lose correlations between them (except the quasi-DNM). Actually, for a higher co-dimensional bifurcation, there are multiple DNMs, but we can obtain the early-warning signals by detecting one of them. In particular, the saddle-node bifurcation is a typical catastrophic bifurcation, whose prediction is of great importance, in contrast to the period-doubling bifurcation that is a non-catastrophic bifurcation. Next, we study the properties

of the Neimark-Sacker bifurcation.

A.1.2 Dynamical network marker near Neimark-Sacker bifurcation point with small noise

We further demonstrate the second case, i.e., the largest eigenvalues are a pair of complex conjugate eigenvalues (including several pairs with the same modulus). For such a case, the critical point is the Neimark-Sacker bifurcation point if the modulus of the largest eigenvalue pair approaches 1. Note that Neimark-Sacker bifurcation is a non-catastrophic bifurcation. We will show that there exist two dominant groups with one common-dominant group for such a case.

Suppose that the dominant eigenvalues are a pair of complex conjugate values $\lambda_1 = a + \mathrm{i}b$ and $\lambda_2 = a - \mathrm{i}b$, with $a^2 + b^2 < 1, \;\; b \neq 0$. The modulus of other eigenvalues are smaller than $a^2 + b^2$. Then for the block corresponding to the dominant eigenvalues, from Eq.(S2) there is

$$\begin{bmatrix} y_1(t+1) \\ y_2(t+1) \end{bmatrix} = \begin{bmatrix} a & b \\ -b & a \end{bmatrix} \begin{bmatrix} y_1(t) \\ y_2(t) \end{bmatrix} + \begin{bmatrix} \xi_1(t) \\ \xi_2(t) \end{bmatrix}$$

where ξ_1, ξ_2 are white noise with mean 0, variance κ_{11}, κ_{22}, and covariance κ_{12}. Then the variances of y_1 and y_2 are as follows

$$\begin{aligned} \mathrm{Var}(y_1) &= \mathrm{Var}(y_1(t+1)) = \mathrm{E}(y_1^2(t+1)) \\ &= \mathrm{E}[(ay_1(t) + by_2(t) + \zeta_1(t))^2] \\ &= a^2\mathrm{Var}(y_1) + b^2\mathrm{Var}(y_2) + 2ab\mathrm{E}(y_1y_2) + \kappa_{11}, \end{aligned}$$

$$\begin{aligned} \mathrm{Var}(y_2) &= \mathrm{Var}(y_2(t+1)) = \mathrm{E}(y_2^2(t+1)) \\ &= \mathrm{E}[(-by_1(t) + ay_2(t) + \zeta_1(t))^2] \\ &= b^2\mathrm{Var}(y_1) + a^2\mathrm{Var}(y_2) - 2ab\mathrm{E}(y_1y_2) + \kappa_{22}, \end{aligned}$$

and the covariance is

$$\begin{aligned}\mathrm{E}(y_1y_2) &= \mathrm{E}(y_1(t+1)y_2(t+1)) \\ &= \mathrm{E}((ay_1(t)+by_2(t)+\zeta_1(t))(-by_1(t)+ay_2(t)+\zeta_1(t))) \\ &= -ab\mathrm{Var}(y_1)+ab\mathrm{Var}(y_2)+(a^2-b^2)\mathrm{E}(y_1y_2)+\kappa_{12}.\end{aligned}$$

By solving the above three equations, we obtain

$$\begin{aligned}\mathrm{Var}(y_1) &= \frac{((1-a^2-b^2)(1-a^2)+2b^2)\kappa_{11}+2ab(1-a^2-b^2)\kappa_{12}+b^2(1+a^2+b^2)\kappa_{22}}{(1-a^2-b^2)((a-1)^2+b^2)((a+1)^2+b^2)} \\ &= \frac{2b^2\kappa_{11}+b^2(1+a^2+b^2)\kappa_{22}}{(1-a^2-b^2)((a-1)^2+b^2)((a+1)^2+b^2)}+\frac{(1-a^2)\kappa_{11}+2ab\kappa_{12}}{((a-1)^2+b^2)((a+1)^2+b^2)}\end{aligned}$$

$$\begin{aligned}\mathrm{Var}(y_2) &= \frac{b^2(1+a^2+b^2)\kappa_{11}-2ab(1-a^2-b^2)\kappa_{12}+((1-a^2-b^2)(1-a^2)+2b^2)\kappa_{22}}{(1-a^2-b^2)((a-1)^2+b^2)((a+1)^2+b^2)} \\ &= \frac{2b^2\kappa_{22}+b^2(1+a^2+b^2)\kappa_{11}}{(1-a^2-b^2)((a-1)^2+b^2)((a+1)^2+b^2)}+\frac{(1-a^2)\kappa_{22}-2ab\kappa_{12}}{((a-1)^2+b^2)((a+1)^2+b^2)}\end{aligned}$$

$$\mathrm{Cov}(y_1,y_2)=\mathrm{E}(y_1y_2)=\frac{-ab\kappa_{11}+(1-a^2+b^2)\kappa_{12}+ab\kappa_{22}}{((a-1)^2+b^2)((a+1)^2+b^2)}.$$

It is clear that both $\lim_{a^2+b^2\to 1}\mathrm{Var}(y_1)=+\infty$ and $\lim_{a^2+b^2\to 1}\mathrm{Var}(y_2)=+\infty$ while $\lim_{a^2+b^2\to 1}\mathrm{Cov}(y_1y_2)$ remain bounded.

For other y_i $(i=3,4,...)$, since their corresponding eigenvalues are not dominant ones, i.e., smaller than a^2+b^2 in the modulus, $\lim_{a^2+b^2\to 1}\mathrm{Var}(y_i)$ $(i=3,4,...)$ is bounded. There are also no correlations between them.

Next, we analyze the variances and correlations for the original variables based on the above results. For the original variables Z, through $Z(k)-\bar{Z}=SY(k)$, we have

$$z_i(k)=s_{i1}y_1(k)+s_{i2}y_2(k)\cdots+s_{in}y_n(k)+\bar{z}_i. \tag{S3}$$

Thus

$$
\begin{aligned}
\mathrm{Var}(z_i) &= s_{i1}^2\mathrm{Var}(y_1) + s_{i2}^2\mathrm{Var}(y_2) + \sum_{k=3}^{n} s_{ik}^2\mathrm{Var}(y_k) \\
&\quad + \sum_{k,m=1,k\neq m}^{n} s_{ik}s_{im}\mathrm{Cov}(y_k, y_m) \\
&= \frac{s_{i1}^2[2b^2\kappa_{11} + b^2(1+a^2+b^2)\kappa_{22}] + s_{i2}^2[2b^2\kappa_{22} + b^2(1+a^2+b^2)\kappa_{11}]}{(1-a^2-b^2)((a-1)^2+b^2)((a+1)^2+b^2)} + K_i' \\
&= \frac{2b^2(s_{i1}^2 + s_{i2}^2)(\kappa_{11}+\kappa_{22})}{(1-a^2-b^2)((a-1)^2+b^2)((a+1)^2+b^2)} + K_i
\end{aligned}
$$

where $K_i' = \sum_{k=3}^{n} s_{ik}^2\mathrm{Var}(y_k) + \sum_{k,m=1,k\neq m}^{n} s_{ik}s_{im}\mathrm{Cov}(y_k, y_m)$ and K_i are bounded values as $a^2 + b^2 \to 1$.

When $s_{i1} \neq 0$ or $s_{i2} \neq 0$, $\lim_{a^2+b^2\to 1} \mathrm{Var}(z_i) \to \infty$.

When both $s_{i1} = 0$ and $s_{i2} = 0$, $\lim_{a^2+b^2\to 1} \mathrm{Var}(z_i) \to K_i$, where K_i is a bounded value.

As for the covariance between two original variables z_i and z_j

$$
\begin{aligned}
&\mathrm{Cov}(z_i, z_j) \\
&= \mathrm{E}((s_{i1}y_1 + \cdots + s_{in}y_n)(s_{j1}y_1 + \cdots + s_{jn}y_n)) \\
&= s_{i1}s_{j1}\mathrm{Var}(y_1) + s_{i2}s_{j2}\mathrm{Var}(y_2) + s_{i1}s_{j2}\mathrm{Cov}(y_1, y_2) + s_{i2}s_{j1}\mathrm{Cov}(y_1, y_2) \\
&\quad + \sum_{k=3}^{n} s_{ik}s_{jk}\mathrm{Var}(y_k) + \sum_{k,m=2,k\neq m}^{n} s_{ik}s_{jm}\mathrm{Cov}(y_k, y_m) \\
&= s_{i1}s_{j1}\left[\frac{2b^2\kappa_{11} + b^2(1 + a^2 + b^2)\kappa_{22}}{(1 - a^2 - b^2)((a-1)^2 + b^2)((a+1)^2 + b^2)} + \frac{(1-a^2)\kappa_{11} + 2ab\kappa_{12}}{((a-1)^2 + b^2)((a+1)^2 + b^2)}\right] \\
&\quad + s_{i2}s_{j2}\left[\frac{2b^2\kappa_{22} + b^2(1 + a^2 + b^2)\kappa_{11}}{(1 - a^2 - b^2)((a-1)^2 + b^2)((a+1)^2 + b^2)} + \frac{(1-a^2)\kappa_{22} - 2ab\kappa_{12}}{((a-1)^2 + b^2)((a+1)^2 + b^2)}\right] \\
&\quad + (s_{i1}s_{j2} + s_{i2}s_{j1})\frac{-ab\kappa_{11} + (1 - a^2 + b^2)\kappa_{12} + ab\kappa_{22}}{((a-1)^2 + b^2)((a+1)^2 + b^2)} \\
&\quad + \sum_{k=3}^{n} s_{ik}s_{jk}\mathrm{Var}(y_k) + \sum_{k,m=2,k\neq m}^{n} s_{ik}s_{jm}\mathrm{Cov}(y_k, y_m) \\
&= \left[\frac{\Delta}{(1 - a^2 - b^2)((a-1)^2 + b^2)((a+1)^2 + b^2)}\right] + C'_{ij} \\
&= \left[\frac{2b^2(s_{i1}s_{j1} + s_{i2}s_{j2})(\kappa_{11} + \kappa_{22})}{(1 - a^2 - b^2)((a-1)^2 + b^2)((a+1)^2 + b^2)}\right] + C_{ij}
\end{aligned}
$$

where $\Delta = (s_{i1}s_{j1}\kappa_{11} + s_{i2}s_{j2}\kappa_{22})2b^2 + (s_{i1}s_{j1}\kappa_{22} + s_{i2}s_{j2}\kappa_{11})b^2(1 + a^2 + b^2)$, and C'_{ij} and C_{ij} are bounded values as $a^2 + b^2 \to 1$.

As for the Pearson's Correlation Coefficient (PCC)

$$
\begin{aligned}
\mathrm{PCC}(z_i, z_j) &= \frac{\mathrm{Cov}(z_i, z_j)}{\sqrt{\mathrm{Var}(z_i)\mathrm{Var}(z_j)}} \\
&= \frac{\left(\frac{2b^2(s_{i1}s_{j1}+s_{i2}s_{j2})(\kappa_{11}+\kappa_{22})}{(1-a^2-b^2)((a-1)^2+b^2)((a+1)^2+b^2)}\right) + C_{ij}}{\sqrt{\left(\frac{2b^2(s_{i1}^2+s_{i2}^2)(\kappa_{11}+\kappa_{22})}{(1-a^2-b^2)((a-1)^2+b^2)((a+1)^2+b^2)} + K_i\right)\left(\frac{2b^2(s_{j1}^2+s_{j2}^2)(\kappa_{11}+\kappa_{22})}{(1-a^2-b^2)((a-1)^2+b^2)((a+1)^2+b^2)} + K_j\right)}}.
\end{aligned}
$$

When $s_{i1} = s_{i2} = s_{j1} = s_{j2} = 0$, $\lim_{a^2+b^2\to 1} \mathrm{PCC}(z_i, z_j) \to P_{ij}$, where P_i is a bounded value.

When $s_{i1}^2 + s_{i2}^2 \neq 0$ and $s_{j1}^2 + s_{j2}^2 = 0$, $\lim_{a^2+b^2\to 1} \mathrm{PCC}(z_i, z_j) \to 0$.

When $s_{i1}s_{j2} \neq 0$, and $s_{i2}^2 + s_{j1}^2 = 0$, $\lim_{a^2+b^2\to 1} \mathrm{PCC}(z_i, z_j) \to 0$.

When $s_{i1}^2 + s_{i2}^2 \neq 0$, and $s_{j1}^2 + s_{j2}^2 \neq 0$,

$$\lim_{a^2+b^2 \to 1} |\text{PCC}(z_i, z_j)| = \lim_{a^2+b^2 \to 1} \frac{|\text{Cov}(z_i, z_j)|}{\sqrt{\text{Var}(z_i)\text{Var}(z_j)}}$$

$$= \frac{|(s_{i1}s_{j1}\kappa_{11} + s_{i2}s_{j2}\kappa_{22}) + (s_{i1}s_{j1}\kappa_{22} + s_{i2}s_{j2}\kappa_{11})|}{\sqrt{((s_{i1}^2\kappa_{11} + s_{i2}^2\kappa_{22}) + (s_{i1}^2\kappa_{22} + s_{i2}^2\kappa_{11}))\left((s_{j1}^2\kappa_{11} + s_{j2}^2\kappa_{22}) + (s_{j1}^2\kappa_{22} + s_{j2}^2\kappa_{11})\right)}}$$

$$= \frac{|s_{i1}s_{j1} + s_{i2}s_{j2}|}{\sqrt{s_{i1}^2 s_{j1}^2 + s_{i2}^2 s_{j2}^2 + s_{i1}^2 s_{j2}^2 + s_{i2}^2 s_{j1}^2}}$$

$$= \frac{|s_{i1}s_{j1} + s_{i2}s_{j2}|}{\sqrt{(s_{i1}s_{j1} + s_{i2}s_{j2})^2 + (s_{i1}s_{j2} - s_{i2}s_{j1})^2}}$$

It is seen that $\lim_{a^2+b^2 \to 1} |\text{PCC}(z_i, z_j)| = 1$ only when $s_{i1}s_{j2} = s_{i2}s_{j1}$. There are following special cases.

- when $s_{i2}s_{j2} \neq 0$ and $s_{i1} = s_{j1} = 0$, $\lim_{a^2+b^2 \to 1} |\text{PCC}(z_i, z_j)| = 1$;
- when $s_{i1}s_{j1} \neq 0$ and $s_{i2} = s_{j2} = 0$, $\lim_{a^2+b^2 \to 1} |\text{PCC}(z_i, z_j)| = 1$;

More generally,

- when $s_{i1}s_{j1} \neq 0$, $s_{i1}^2 >> s_{i2}^2$ and $s_{j1}^2 >> s_{j2}^2$ (i.e., $\frac{s_{i2}}{s_{i1}} \approx 0$ and $\frac{s_{j2}}{s_{j1}} \approx 0$),

  $\lim_{a^2+b^2 \to 1} |\text{PCC}(z_i, z_j)| = 1$.

  It means that when two variables z_i, z_j are much more related to y_1 than to y_2, generally they belong to y_1-related dominant group.

- when $s_{i2}s_{j2} \neq 0$, $s_{i2}^2 >> s_{i1}^2$ and $s_{j2}^2 >> s_{j1}^2$ (i.e., $\frac{s_{i1}}{s_{i2}} \approx 0$ and $\frac{s_{j1}}{s_{j2}} \approx 0$),

  $\lim_{a^2+b^2 \to 1} |\text{PCC}(z_i, z_j)| = 1$.

  It means that when two variables z_i, z_j are much more related to y_2 than to y_1, generally they belong to y_2-related dominant group.

Therefore, there are the following critical results (also see Fig.S2).

- If $s_{i1}, s_{j1} \neq 0$ and $s_{i2}, s_{j2} = 0$, i.e., both z_i, z_j are only related to y_1, then $|\text{PCC}(z_i, z_j)| \to 1$ as $a^2 + b^2 \to 1$.

  More generally, if $s_{i1}, s_{j1} \neq 0$, $s_{i1}^2 >> s_{i2}^2$ and $s_{j1}^2 >> s_{j2}^2$, then $|\text{PCC}(z_i, z_j)| \approx 1$ as $a^2 + b^2 \to 1$.

- If $s_{i2}, s_{j2} \neq 0$ and $s_{i1}, s_{j1} = 0$, i.e., both z_i, z_j are only related to y_2, then $|\text{PCC}(z_i, z_j)| \to 1$ as $a^2 + b^2 \to 1$.

  More generally, if $s_{i2}, s_{j2} \neq 0$, $s_{i2}^2 >> s_{i1}^2$ and $s_{j2}^2 >> s_{j1}^2$, then $|\text{PCC}(z_i, z_j)| \approx 1$ as $a^2 + b^2 \to 1$.

- if $s_{i1}, s_{j2} \neq 0$ and $s_{i2}, s_{j1} = 0$, i.e., z_i is related to y_1 and z_j is related to y_2, then $\Delta = 0$ and $\text{PCC}(z_i, z_j) \to 0$, as $a^2 + b^2 \to 1$;

- if $s_{i1}, s_{j1}, s_{j2} \neq 0$ and $s_{i2} = 0$, i.e., z_i is only related to y_1 and z_j is related to both y_1 and y_2, then $\text{PCC}(z_i, z_j) \to a \in (-1, 1)$, as $a^2 + b^2 \to 1$;

- if $s_{i1}, s_{j1}, s_{i2}, s_{j2} \neq 0$, i.e., both z_i, z_j are related to y_1 and y_2, $\text{PCC}(z_i, z_j) \to b \in (-1, 1)$ as $a^2 + b^2 \to 1$.

- if $s_{i1} = 0$ and $s_{i2} = 0$, i.e., z_i has nothing to do with y_1 and y_2, then

  $s_{j1} \neq 0$ or $s_{j2} \neq 0$, i.e., z_j is related to y_1 or y_2, $\text{PCC}(z_i, z_j) \to 0$, as $a^2 + b^2 \to 1$;

  $s_{j1} = 0$ and $s_{j2} = 0$, i.e., z_j has nothing to do with y_1 and y_2, and $\text{PCC}(z_i, z_j)$ is a bounded value in $(-1, 1)$, as $a^2 + b^2 \to 1$.

Therefore, there are two dominant groups respectively related to y_1 and y_2 (see Fig.S2). The PCC between a y_1-related member z_i and a y_2-related member z_j is either 1 (if z_i or z_j is related to y_1 or y_2, respectively) or bounded value (if z_i and z_j are related to y_1 and y_2 simultaneously). To summarize and state these results clearly, we make the following definitions.

- Dominant group 1: contains variables z_i where $s_{i1} \neq 0$ and $s_{i2} = 0$, i.e., variables are only related to y_1.
- Dominant group 2: contains variables z_i where both $s_{i1} = 0$ and $s_{i2} \neq 0$, i.e., variables are only related to y_2.
- Common-dominant group: contains variables z_i where $s_{i1} \neq 0$ and $s_{i2} \neq 0$, i.e., variables related to y_1 and y_2 simultaneously.
- Non-dominant group: contains variables z_i where $s_{i1} = 0$ and $s_{i2} = 0$, i.e., variables have no relation with y_1 and y_2.

Theorem 2 *We consider a stochastically perturbed linearized system for Eq.(S1). When P approaches the Neimark-Sacker bifurcation point, the following results hold (also see Fig.S2).*

- *If both z_i and z_j are in the same dominant group 1 or 2, then*

$$|\mathrm{PCC}(z_i, z_j)| \to 1,$$

  while $\mathrm{SD}(z_i) \to \infty$ *and* $\mathrm{SD}(z_j) \to \infty$;

- *if z_i is in dominant group 1 and z_j in dominant group 2, then*

$$\mathrm{PCC}(z_i, z_j) \to 0,$$

  while $\mathrm{SD}(z_i) \to \infty$*, and* $\mathrm{SD}(z_j) \to \infty$*;*

- *if z_i is in a dominant group (including dominant 1 or 2, common-dominant group) and z_j is in the non-dominant group, then*

$$\mathrm{PCC}(z_i, z_j) \to 0,$$

  while $\mathrm{SD}(z_i) \to \infty$ *and* $\mathrm{SD}(z_j)$ *approaches a bounded value;*

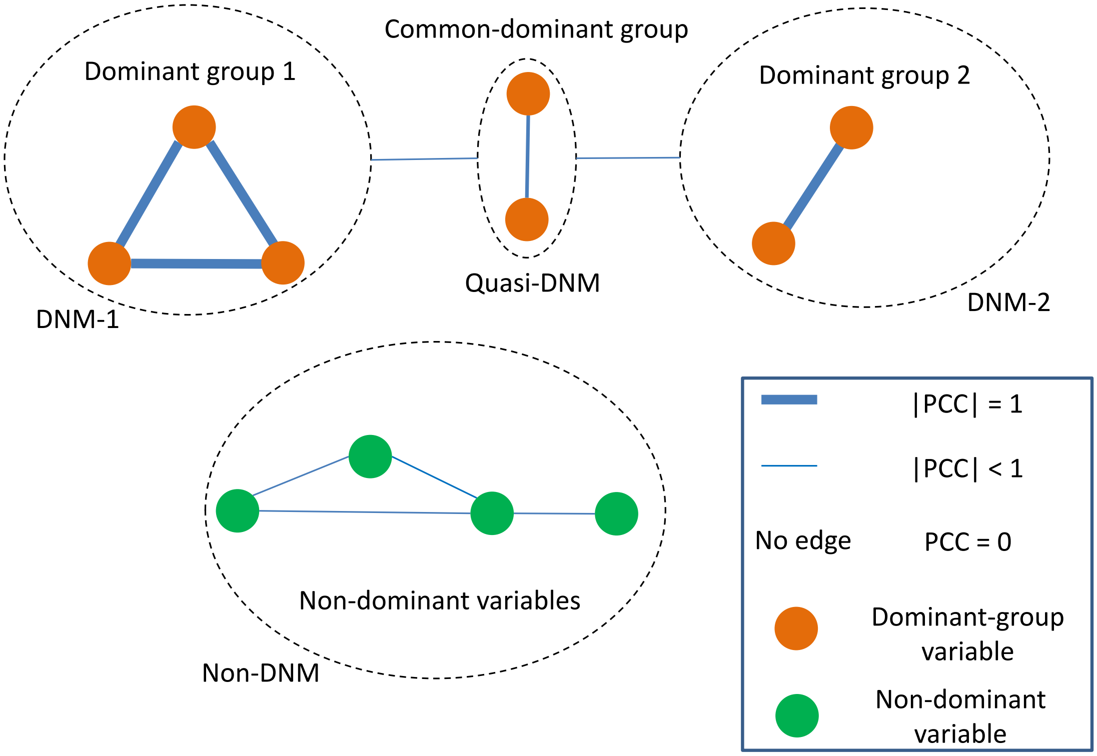

Figure S2: | **A sketch for the case of two dominant groups (two DNMs) with one common-dominant group (one quasi-DNM) when the system approaches the critical point.** With the assumption of a pair of conjugate complex eigenvalues, there two dominant groups and one one common-dominant group in a network just before a critical transition, where the orange nodes represent variables inside the dominant groups, while green nodes are outside (belong to non-dominant group). Each edge represents the correlation between two variables. It can be seen that among orange nodes in a same dominant group 1 or 2, the correlations are strong. However, between dominant group 1 (or 2) and the common-dominant group, any two nodes show relatively weak correlation. There is no correlation between dominant group 1 and dominant group 2, and between a dominant-group member and a non-dominant variable. Note that, if the system is not near the critical point, generally there are edges between nodes of the dominant variables and non-dominant variables, which are all in one network.

- *if* z_i *is in the common dominant group, and* z_j *is in dominant group* k $(k = 1, 2)$ *or the common dominant group, then* $|\mathrm{PCC}(z_i, z_j)|$ *approaches a constant less than 1, while* $\mathrm{SD}(z_i) \to \infty$*, and* $\mathrm{SD}(z_j) \to \infty$*;*

- *if neither* z_i *nor* z_j *is in the dominant group, then* $|\mathrm{PCC}(z_i, z_j)|$ *approaches a constant*

less than 1, while both $\mathrm{SD}(z_i)$ *and* $\mathrm{SD}(z_j)$ *approach bounded values;*

where PCC is the Pearson's correlation coefficient and SD is the standard deviation.

Remark 1 *When both* z_i *and* z_j *are in the common dominant group, if their relations to* y_1 *are much more stronger than that to* y_2*, then generally* z_i *and* z_j *are considered in dominant group 1. Contrarily, if the relations of* z_i *and* z_j *to* y_2 *are much more stronger than that to* y_1*, then generally* z_i *and* z_j *are considered in dominant group 2.*

Thus, the members in the common dominant group are related to y_1 and y_2 in a similar way, e.g., z_i and z_j approximately satisfy $s_{i1}/s_{i2} \approx s_{j1}/s_{j2}$, and then $\lim_{a^2+b^2 \to 1} |\mathrm{PCC}(z_i, z_j)| \approx 1$. Note that the theorem holds for a linearized system.

A.1.3 Dynamical network marker for nonlinear systems near critical transition point with small noise

We presented the critical properties of the linearized system for Eq.(S1). However, for a nonlinear system Eq.(S1) approaching the bifurcation point (the saddle-node or period-doubling bifurcation point) or tipping point, we can observe directly obtain the properties of DNM based on Theorem 1 as the following remark.

Remark 2 *For nonlinear case Eq.(S1) near a bifurcation point (the saddle-node or period-doubling bifurcation point), the dynamical behavior has the same tendency as that of the linearized case, that is, when the system is approaching to the bifurcation point, both indices* SD *and* $|\mathrm{PCC}|$ *in the DNM increase sharply, while* $|\mathrm{PCC}|$ *between DNM and other non-DNM molecules decreases rapidly, i.e.,*

1. *If both* z_i *and* z_j *are in the DNM, then* $\mathrm{PCC}(z_i, z_j)$ *increases, while* $\mathrm{SD}(z_i)$ *and* $\mathrm{SD}(z_j)$ *drastically increase;*

2. *if* z_i *is in the DNM but* z_j *is not, then* $\mathrm{PCC}(z_i, z_j)$ *decreases, while* $\mathrm{SD}(z_i)$ *drastically increases, and there is no significant change for* $\mathrm{SD}(z_j)$*;*

3. *if neither* z_i *nor* z_j *is in the DNM, then there are no significant changes on* $\mathrm{PCC}(z_i, z_j)$*,* $\mathrm{SD}(z_i)$ *and* $\mathrm{SD}(z_j)$*.*

For a nonlinear system Eq.(S1) approaching the Neimark-Sacker bifurcation point, we can obtain the properties of DNMs based on Theorem 2 as the following remark. There are DNM 1, DNM 2, quasi-DNM and non-DNM respectively corresponding to dominant group 1, dominant group 2, common-dominant group and non-dominant group.

Remark 3 *For nonlinear case Eq.(S1) near a Neimark-Sacker bifurcation point, the dynamical behavior has the same tendency as that of the linearized case, that is, when the system is approaching to the bifurcation point, both indices* SD *and* $|\mathrm{PCC}|$ *in DNM* k $(k = 1, 2)$ *increase sharply, However, for the three cases, i.e., any two members between DNM 1 and DNM 2, between DNM* k $(k = 1, 2)$ *and non-DNM molecules, and between quasi-DNM and non-DNM molecules,* $|\mathrm{PCC}|$ *decreases rapidly. On the other hand, for the other two cases, i.e., any two members in quasi-DNM, and between DNM* k $(k = 1, 2)$ *and quasi-DNM,* $|\mathrm{PCC}|$ *has no significant change. i.e.,*

1. *If both* z_i *and* z_j *are in DNM 1 (or DNM 2), then* $\mathrm{PCC}(z_i, z_j)$ *increases, while* $\mathrm{SD}(z_i)$ *and* $\mathrm{SD}(z_j)$ *drastically increase;*

2. *if* z_i *is in DNM 1 (or DNM 2, or quasi-DNM) but* z_j *belongs to non-DNM, then* $\mathrm{PCC}(z_i, z_j)$ *decreases, while* $\mathrm{SD}(z_i)$ *drastically increases, and there is no significant change for* $\mathrm{SD}(z_j)$*;*

3. *if* z_i *is in DNM 1 and* z_j *is in DNM 2, then* $\mathrm{PCC}(z_i, z_j)$ *decreases, while both* $\mathrm{SD}(z_i)$ *and* $\mathrm{SD}(z_j)$ *drastically increase;*

4. *if z_i is in DNM 1 (or DNM 2) and z_j is in quasi-DNM, then there is no significant changes on* $\mathrm{PCC}(z_i, z_j)$*, while both* $\mathrm{SD}(z_i)$ *and* $\mathrm{SD}(z_j)$ *drastically increase;*

5. *if both z_i and z_j are in quasi-DNM, then there is no significant changes on* $\mathrm{PCC}(z_i, z_j)$*, while* $\mathrm{SD}(z_i)$ *and* $\mathrm{SD}(z_j)$ *drastically increase;*

6. *if neither i nor j is in any DNM, then there are no significant changes on* $\mathrm{PCC}(z_i, z_j)$*,* $\mathrm{SD}(z_i)$ *and* $\mathrm{SD}(z_j)$*.*

Note that for 5 in Remark 3, the members in the common dominant group are related to y_1 and y_2 in a similar way, e.g., z_i and z_j approximately satisfy $s_{i1}/s_{i2} \approx s_{j1}/s_{j2}$, and thus $\lim_{a^2+b^2 \to 1} |\mathrm{PCC}(z_i, z_j)| \approx 1$. As indicated in the theoretical results, there is only one DNM for any co-dimension-one bifurcation except Neimark-Sacker bifurcation which corresponds to two DNMs with one quasi-DNM. For these two DNMs, they are all with strong SDs, but lose correlations between them (except the quasi-DNM). Actually, for a higher co-dimensional bifurcation, there are multiple DNMs, but we can obtain the early-warning signals by detecting one of them. In particular, the saddle-node bifurcation is a typical catastrophic bifurcation, whose prediction is of great importance, in contrast to the period-doubling bifurcation that is a non-catastrophic bifurcation.

Based on above results, by using the data of either multiple-samples or time-course high-throughput data for complex biological processes, we can identify the corresponding DNMs (or DNBs: dynamical network biomarkers) based on the above conditions, e.g., by the algorithm in the following section D or the algorithm described for DNB (*15, 17*). Clearly, this approach is a model-free method, and DNM can be obtained only based on the data. Note that sample data without time points can also be used to construct DNM based on the above conditions.

Critical slowing-down (CSD) (*16*) for single variables has been considered as a leading indicator to predict the critical transitions provided that the system is fluctuated by small noise,

which assumes the linear restoring force, i.e., small noise. However, CSD mainly characterizes the dynamics of single-variables, in contrast to DNM which characterizes the dynamics of multi-variables or a network. Actually, for a single-variable system, DNM is equivalent to the principle of CSD.

A.2 Three states during a critical transition process

During a state transition, the dynamics of the system can be divided as three stages, as shown in Fig.S1. Before-transition state corresponds to a stable equilibrium. In bio-medical systems, it generally corresponds to a "normal state" or a stable period that the disease is under control. Pre-transition state is the limit of the before-transition state. In bio-medical systems, it represents a "pre-disease state" just before the critical transition to the disease state. After-transition state corresponds to another stable equilibrium. In bio-medical systems, it represents a badly ill stage or "disease state", and is usually difficult to return to the before-transition state even by big perturbations.

As shown in Table S1, for one-dimension system, the before-transition state and pre-transition state are near each other with the similar values, whereas the after-transition state is significantly different from the above two states. On the other hand, the variation (low) of the before-transition state is significantly different from that (high) of the pre-transition state, but may be similar to that (low) of the after-transition state. For multi-dimension system, the before-transition state and pre-transition state are near each other with the similar values, whereas the after-transition state is significantly different from the above two states. On the other hand, the score (low) of the dynamical network marker of the before-transition state is significantly different from that (high) of the pre-transition state, but may be similar to that (low) of the after-transition state.

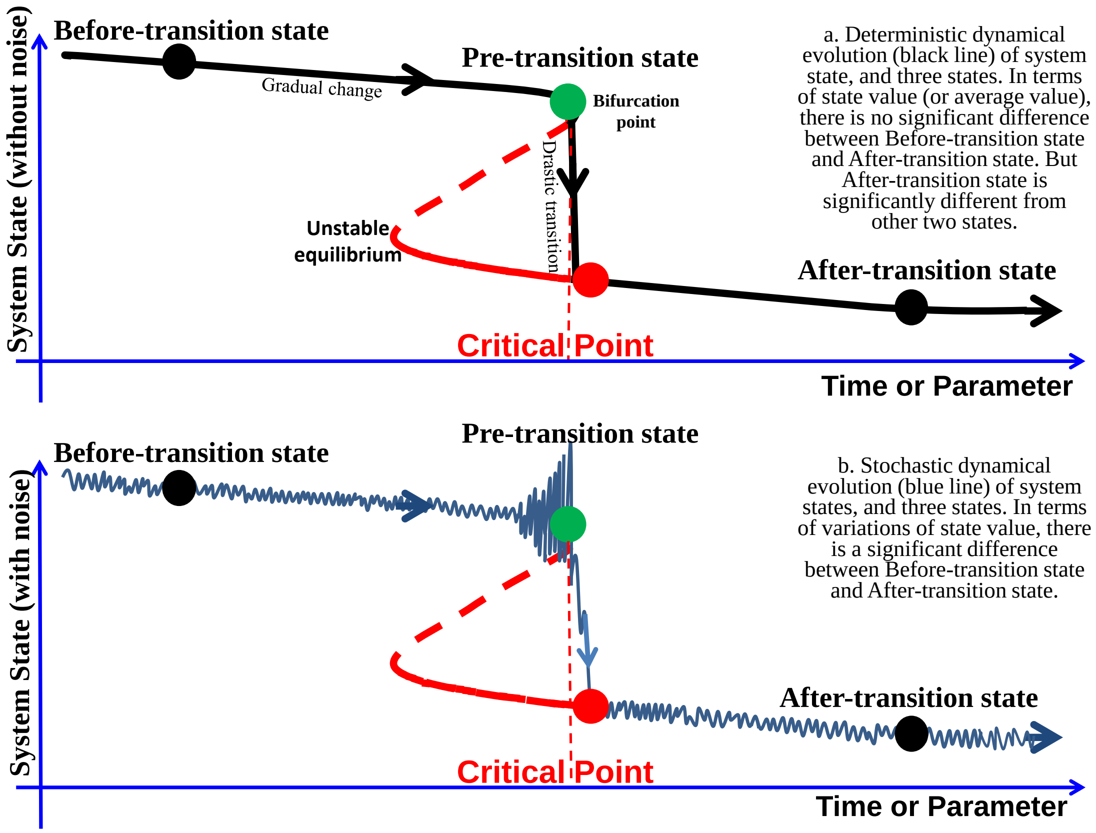

Figure S3: | **Definition of three states in a transition process.** Before-transition state is a stable equilibrium. Pre-transition state is the limit of the Before-transition state. After-transition state is another stable equilibrium. **(a)**. Deterministic dynamical evolution (black line) of system state with time or parameter. **(b)**. Stochastic dynamical evolution (blue line) of system states with time or parameter. In terms of state value (i.e., when it is a deterministic system or we only consider average value of a stochastic system), there is no significant difference between before-transition state and pre-transition state. But after-transition state is significantly different from other two states. In terms of variations of state value (when it is fluctuated by noise or it is a stochastic system), there is a significant difference between the before-transition state and the pre-transition state. In a real system, the system is always fluctuated by noise, and thus the process of a state transition can be characterized by three states. Also see Table S1 for the features of the three states in a process of state transition. The green circle represents the pre-transition or critical state, while the red one is the state immediately after the transition. We aim to detect the pre-transition state or green circle, rather than the red circle.

A.3 Distribution embedding near critical transition point with big noise by moment expansion

Note that we do not consider the flickering phenomenon in this paper. In other words, we assume only to have the observed data near the original stable state before the critical transition

Table S1. Features of three states in a process of state transition with small noise

One-dimension System	Before-transition state	Pre- transition state	After-transition state
Average of state values	Similar level	Similar level	Different level
Variation of state values	Low	High	Low
Detection methods for small noise		SD, Autocorrelation, Skewness, etc.	
**Multi-dimension system**	**Before-transition state**	**Pre- transition state**	**After-transition state**
Average of variables	Similar level	Similar level	Different level
Correlations and variations of state values	Low	High	Low
Detection methods for small noise		Dynamical network marker, cross-correlation, etc.	

Thus, based on the above features, we can distinguish the three states when the system is fluctuated with small noise.

to another state. Thus, the probability distribution from the observed data is the conditional distribution for the whole system (e.g., in contrast to the stationary probability distribution) due to no observed data on the after-transition state.

When the system is fluctuated by big noise, the critical point is far earlier than the bifurcation point, which may make critical slowing-down principle fail. However, we can transform the stochastic system into moment equations, a set of ordinary differential equations (ODEs) with moments as variables, that is, the mean, variance, skewness and so on, and thus reduce the level of the original noise. A set of moments correspond to a probability distribution, and such a transformation is actually to convert the state dynamics into the distribution dynamics. In the following, we explain such a procedure based on dynamical systems theory.

Generally, a dynamical system with big noise can be expressed by the following stochastic

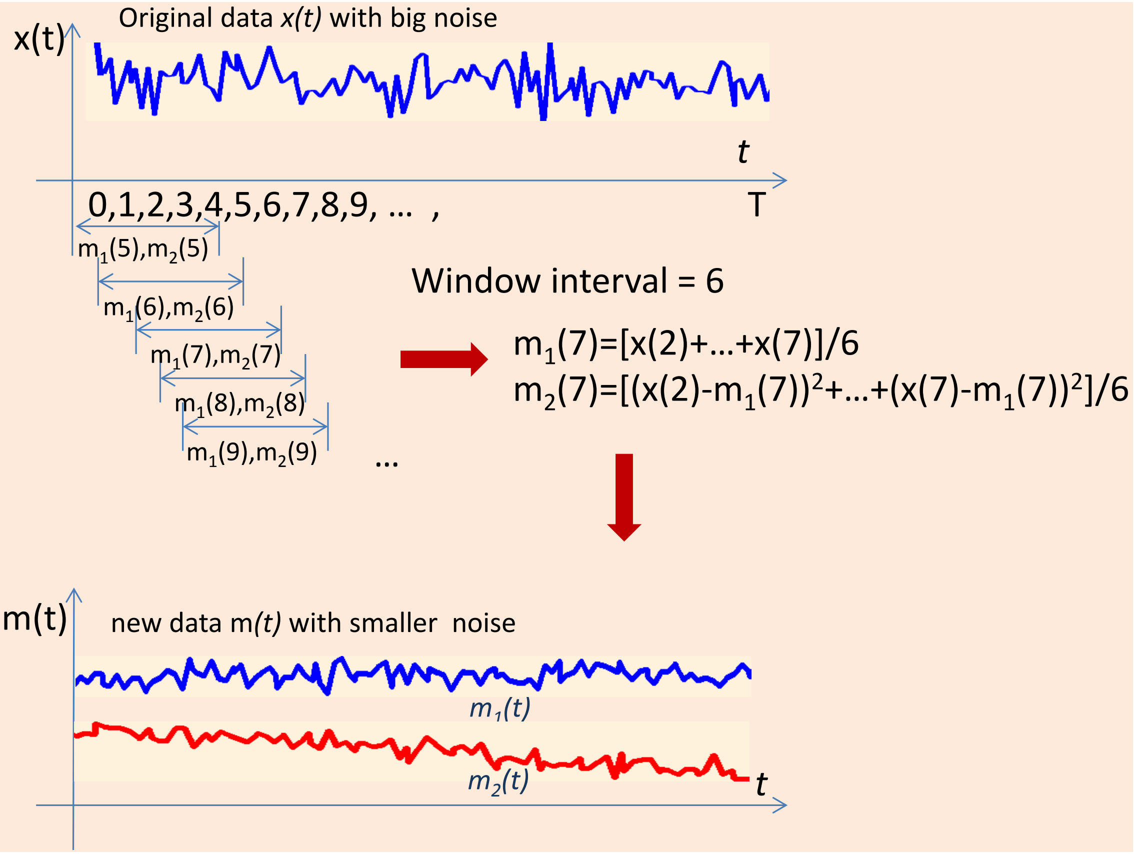

Figure S4: | **A sketch for sliding window.**

differential equation

$$\frac{\mathrm{d}x(t)}{\mathrm{d}t} = f(x(t)) + \eta_{big}(t), \tag{S4}$$

where $f(x(t)) = (f_1(x(t)), ..., f_n(x(t)))$ are nonlinear functions, state variables are $x(t) = (x_1(t), ..., x_n(t))$, and noises are $\eta_{big}(t) = (\eta_{big\_1}(t), ..., \eta_{big\_n}(t))$ with mean $\langle \eta_{big\_i}(t) \rangle = 0$ and covariance $\langle \eta_{big\_i}(t), \eta_{big\_j}(t) \rangle = \sigma_{big\_i,j}$. Here the angle brackets $\langle \cdot \rangle$ is the operator for calculating the average.

Then, we can approximate system (S4) by the following moment evolution equation *(11)* with moment expansion to the kth order:

$$\frac{\mathrm{d}m(t)}{\mathrm{d}t} = g(m(t)) + \eta_{small}(t) \tag{S5}$$

where $g(m(t)) = (g_1(m(t)), ..., g_N(m(t)))$ are nonlinear moment functions derived from $f(x(t))$, and moment variables are $m(t) = (m_1(t), ..., m_N(t))$. Due to truncation to the k-th order of moments, the error functions are $\eta_{small}(t) = (\eta_{small\_1}(t), ..., \eta_{small\_N}(t))$, which can be taken as noise terms. $m_i(t)$ is a moment, and N is the total number of the moments up to the k-th order. In particular, if expanding the moments to $k = 2$, then $N = n(n + 3)/2$, where the moment variables $m_1(t)$ are means (the first order moment of variable x_i, i.e., $\langle x_i \rangle$) and $m_2(t)$ are covariances (the second order central moments of variables x_i and x_j, i.e., $\langle (x_i - \langle x_i \rangle)(x_j - \langle x_j \rangle) \rangle$) of $x(t)$. Actually, to approximate the original stochastic dynamics or minimize the error terms $\eta_{small}(t)$ by finite order moment equations (S5), many sophisticated schemes to truncate moments, such as moment closure (*12, 13*), have been proposed. By this moment-system with smaller noise, we can directly use DNM to detect the critical transition, where the critical point is not the bifurcation point of the original system (S4) but the one of (S5). Note that any probability distribution can be represented or expanded by Gram-Charlier or Edgeworth series (*26*) in terms of moments $m(t)$. Hence, a set of moments represent one probability distribution, i.e., this moment-system represents the dynamics of the state probability-distribution rather than the state dynamics of the original system $x(t)$, and thus the critical point of the moment-system (S5) corresponds to the drastic change of the probability-distribution rather than the drastic change of the state $x(t)$. In other words, different from the critical state-transition of the deterministic system in terms of $x(t)$, the transition of the stochastic system (S5) or (S6) in terms of $m(t)$ is the critical distribution-transition.

For a linear system, (S4) can even be exactly expressed by (S5) with the moment expansion up to the second order, i.e., $k = 2$. For this case, there is no error, i.e., the noise is reduced to zero, $\eta_{small}(t) = \{0, ..., 0\}$. For a nonlinear system, if x follows Gaussian distribution, (S4) can also be exactly expressed by (S5) with $k = 2$ and zero error.

For a general nonlinear stochastic system, with moment expansion to an infinite order (*11*),

i.e., as $k \to \infty$, the dynamics of system (S4) can be expressed by (S6) in an exact manner, which becomes a deterministic system with zero error or noise.

$$\frac{\mathrm{d}M(t)}{\mathrm{d}t} = F(M(t)), \tag{S6}$$

where $F(M(t)) = (g_1(M(t)), g_2(M(t)), ...)$ are nonlinear moment functions, and moment variables are $M(t) = (m_1(t), m_2(t), ...)$. The error functions are reduced to zero, i.e., $\eta_{small}(t) = (0, 0, ...)$. Also see the intuitive explanation in Figure 1 in the main text.

In other words, it is expected that, the higher the order of moment expansion is, the more accurate the resulting dynamics (S5) would be to that of the original system (S4) in terms of the distribution, and thus the smaller the noises or error terms are. This result gives the theoretical basis to reduce the noise level by increasing the dimension of the original system. In particular, the moment system corresponds to the distribution dynamics, i.e., a set of moments represent one distribution. Thus, (S5) or (S6) can be also viewed as the transformation from state dynamics with big noise to distribution dynamics with small noise. Note that we can only observe the data in the original state before the transition, and have no information on the state after the transition, i.e., no flickering. Thus, the observed probability distribution is the conditional distribution. Also note that in real situations, we only have observed data, and do not need the above analytical implementations. Next we will first describe the implication of the critical transition for the moment-system, and then give the detail procedure to construct the synthetic data in a higher-dimensional space from the observed original data.

Next, we specifically derive the moment evolution equations by expanding the moments to the second order, i.e., $k = 2$. Let the first-order moment (or mean) be $u = \{u_1, ..., u_n\}$ with $u_i = \langle x_i \rangle$, and the second-order moment (or covariance) be $v = \{v_{ij}\}_{i,j=1,2,...,n}$ with $v_{ij} = \langle (x_i - u_i)(x_j - u_j) \rangle$. Then the moment equations are the following deterministic system

(*11*)

$$\frac{\mathrm{d}u_i(t)}{\mathrm{d}t} = g_i(u(t), v(t)), \quad i = 1, 2, ..., n, \tag{S7}$$

$$\frac{\mathrm{d}v_{ij}(t)}{\mathrm{d}t} = g_{ij}(u(t), v(t)), \quad i, j = 1, 2, ..., n. \tag{S8}$$

where

$$g_i(u(t), v(t)) = \langle f_i(x(t)) \rangle, \tag{S9}$$

$$g_{ij}(u(t), v(t)) = \langle (x_i(t) - u_i(t)) f_j(x(t)) + (x_j(t) - u_j(t)) f_i(x(t)) \rangle + \sigma_{ij}. \tag{S10}$$

Therefore, the original stochastic system (S4) is transformed to a deterministic system (S7)-(S8).

If the original system is linear, that is, $f(x) = Ax + B$, where $A = (A_{ij})$ is an $n \times n$ constant matrix and $B = (B_i)$ is a constant n vector, then obviously we can analytically derive the moment system (S7)-(S8) directly, due to

$$\begin{aligned}
&g_i(u(t), v(t)) \\
&= \langle f_i(x(t)) \rangle \\
&= \langle \sum_{k=1}^{n} A_{ik} x_k + B_i \rangle = \sum_{k=1}^{n} A_{ik} \langle x_k \rangle + B_i = \sum_{k=1}^{n} A_{ik} u_k + B_i,
\end{aligned}$$

$$\begin{aligned}
&g_{ij}(u(t), v(t)) \\
&= \langle (x_i(t) - u_i(t)) f_j(x(t)) + (x_j(t) - u_j(t)) f_i(x(t)) \rangle + \sigma_{ij} \\
&= \langle (x_i(t) - u_i(t)) (\sum_{k=1}^{n} A_{jk} x_k + B_j) + (x_j(t) - u_j(t)) (\sum_{k=1}^{n} A_{ik} x_k + B_i) \rangle + \sigma_{ij} \\
&= \sum_{k=1}^{n} A_{jk} (\langle x_k x_i \rangle - u_k u_i) + \sum_{k=1}^{n} A_{ik} (\langle x_k x_j \rangle - u_k u_j) + \sigma_{ij} \\
&= \sum_{k=1}^{n} A_{jk} v_{ki} + \sum_{k=1}^{n} A_{ik} v_{kj} + \sigma_{ij}.
\end{aligned}$$

Thus, the original system can analytically expressed by the first-order moments u and the second-order moments v.

However, it the original system is nonlinear, the deterministic system (S7)-(S8) is generally unclosed with the first and second order moments. That is, in the expressions (S9) and (S10) there are usually involved with high-order moments (with the third or higher order moments). To circumvent this problem, the approximation methods, such as moment-closure (*41*), are used to truncate moments up to the second order, thereby making (S7)-(S8) closed in terms of the first and second order moments. Due to such an approximation, there are additional error or noise terms in g_i and g_{ij} of (S7)-(S8), as described in (S5). Note that we can expand the original system also by binomial moment series (*27*) in a similar way.

A.4 Critical state-transition and critical distribution-transition

Note that we do not consider the flickering phenomenon in this paper, i.e., we assume only to have the observed data near the original stable state before the critical transition to another state, and based on such observed data, to detect the early-warning signals of the critical transition. Thus, the probability distribution from the observed data is the conditional distribution for the whole system due to no observed data on the after-transition state.

By this moment-system (S5) or (S6) with smaller noise, we can directly use DNM to detect the critical transition, where the critical point is not the bifurcation point of the original system (S4) but the one of (S5) or (S6). As described in Supplementary Information B, any probability-distribution can be represented or expanded by Gram-Charlier or Edgeworth series in terms of moments $m(t)$. Hence, the moment-system represents the dynamics of the state probability-distribution rather than the state dynamics of the original system, and thus the critical point of (S5) or (S6) corresponds to the drastic change of the probability-distribution for state $x(t)$ (i.e., distribution-transition) rather than the drastic change of the state $x(t)$ (i.e., state-transition). In

other words, the bifurcation point of the moment-system (S5) $m(t)$ is the critical "distribution-transition" point (for a stochastic system with noise), whereas the traditional bifurcation point of the original state-system (S4) $x(t)$ is the "state-transition" point (for a deterministic system without noise) (see Supplementary Information B and Fig.S11). Thus, the critical transition in this paper implies the critical distribution-transition, which is the generalization of the traditional state-transition (see Fig.S11).

A.5 Constructing time-course data of moments in a higher-dimensional space from original data

From the observed original data $x(t)$ of state variables, we can construct the time-course data of moment variables $m(t)$ in a higher-dimensional space by simply using a sliding-window scheme, as shown in Fig.S2. In particular, for $k = 2$ with the window interval τ (an integer), the time course data for the first order moment of variable $x_i(t)$ with $i = 1, ..., n$ can be obtained by

$$\langle x_i(t) \rangle = \frac{\sum_{k=t-\tau+1}^{t} x_i(k)}{\tau},$$

where there are n first order moments (or means) and $t \geq \tau$. On the other hand, for the second central moment of variable $x_i(t)$ and $x_j(t)$ with $i, j = 1, ..., n$, we have

$$\langle (x_i(t) - \langle x_i(t) \rangle)(x_j(t) - \langle x_j(t) \rangle) \rangle = \frac{\sum_{k=t-\tau+1}^{t} x_i(k) x_j(k)}{\tau} - \frac{\sum_{k=t-\tau+1}^{t} x_i(k)}{\tau} \frac{\sum_{k=t-\tau+1}^{t} x_j(k)}{\tau},$$

where there are $n(n+1)/2$ second order moments (or covariances) due to their symmetry of the covariances.

From such a procedure, the original n-dimension time-course data are transformed into $n(n+3)/2$-dimension time-course data. Depending on the observed system, if higher order moments ($k > 2$) are required so as to make the noise sufficiently small, we can obtain their time-course data in a similar way. Thus, instead of the original data with big noise, we can

use DNM to analyze the transformed higher-dimensional data with the reduced noise level for detecting the early-warning signals of the critical transition.

B One-dimensional example

In this section, we illustrate the DNM method as well as the critical distribution transition by using an one-dimensional example.

B.1 Moment-system

An one-dimensional state system $x(t)$ is represented as follows.

$$\frac{\mathrm{d}x(t)}{\mathrm{d}t} = -p + 3x(t) - x(t)^3 + \eta(t), \tag{S11}$$

where η is a white noise with zero mean, i.e., $\langle \eta \rangle = 0$, and the variance (or amplitude) $\langle \eta^2 \rangle = \sigma$.

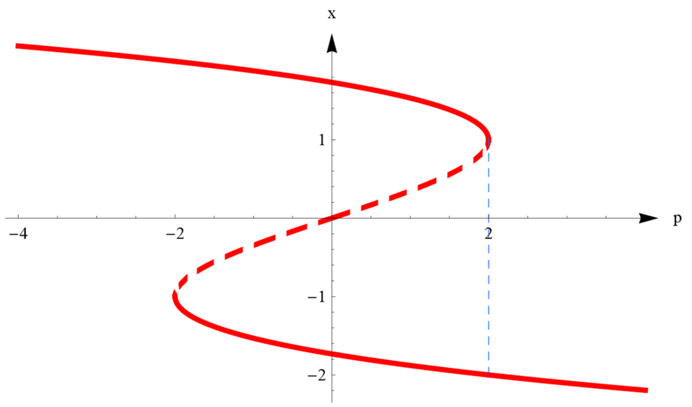

Figure S5: | **The state transition in system of Eq.(S11) without noise** The sketch presents the state transition in the deterministic system (S11). The solid curves represent the stable equilibria and the dashed curve stands for the unstable equilibria. With the increase of the parameter p, the transition occurs near $p = 2$ with small noise.

By analyzing Eq.(S11), we find that when the system is under a small noise ($\sigma = 0.1$), there is a bifurcation point around $p = 2$ (see Fig.S5). As shown in Fig.2 in the main text, the traditional CSD-based criteria, i.e., SD and AR can signal the critical transition since the critical

point is near the bifurcation point. However, when the noise level increases to $\sigma = 1.5$, both SD and AR fail to signal the imminent transition, which occurs much earlier than that of the original system under small noise. Therefore, we use the moment-expanding scheme to "make big noise smaller" so that the DNM-based method works again. Actually, when the system is under big noise, the critical point is far ahead the bifurcation point, comparing with the case with small noise, where the critical point is close to the bifurcation point (see Fig.2 in the main text). Generally, the big noise actually makes the critical transition occurring earlier.

Let $E(x)$ represent the expectation value of x. Through the process of moment closure, i.e.,

$$\mathrm{E}(x^3) = 3m_1m_2 + m_1^3, \tag{S12}$$

$$\mathrm{E}(x^4) = 3m_2^2 + 6m_1^2m_2 + m_1^4, \tag{S13}$$

we get the 2-dimensional moment equation of Eq.(S11) as follows

$$\frac{\mathrm{d}m_1(t)}{\mathrm{d}t} = -p + 3m_1(t) - 3m_1(t)m_2(t) - m_1^3(t) + \xi_1(t), \tag{S14}$$

$$\frac{\mathrm{d}m_2(t)}{\mathrm{d}t} = 6m_2(t) - 6m_1(t)^2m_2(t) - 6m_2(t)^2 + \sigma + \xi_2(t), \tag{S15}$$

where m_1 is the mean (the 1st-order moment) and m_2 is the variance (the 2nd-order moment), σ is the amplitude of original white noise η, and ξ_i is a small noise generated from moment closure.

For 2-dimensional system (S14, S15), the DNM-based method works again due to the reduced noise level. We respectively computed the SDs and ARs for the first-order moment (m_1) and the second-order moment (m_2) in system (S14, S15). The prediction results by DNM is given as Fig.2g-i in the main text.

B.2 Critical state-transition represented by states, and critical distribution-transition by moments

Generally, the probability distribution of $x(t)$ can be represented or expanded by Gram-Charlier or Edgeworth series (*26*) in terms of moments $m(t)$ or cumulants, i.e., the probability distribution of $x(t)$ can be expressed as $\phi(x(t)) = P_{rob}(x(t)) = f(x(t); m(t))$. With the finite moments or cumulants (i.e., the finite terms of Gram-Charlier series), the Gram-Charlier series is the approximation to the original probability distribution, but a Gaussian distribution can be exactly represented by the Gram-Charlier series with first and second moments or cumulants. Note that the probability distribution here is not the stationary distribution in the whole space but the probability distribution in the basin of the original stable equilibrium. Also it is the approximation of the real probability distribution because of the finite moments or moment closure. Thus, the bifurcation for the moment-system with the order-two moments will result in the drastic change of the partial distribution for the Gaussian part, i.e., will result in the distortion of the Gaussian partial-distribution.

Thus the critical point or bifurcation point of the moment-system (S14)-(S15) corresponds to the drastic change of this probability distribution rather than the drastic change of the state $x(t)$. In other words, different from the critical state-transition of the deterministic system in terms of $x(t)$, the transition of the stochastic system (S14)-(S15) in terms of $m_1(t), m_2(t)$ is the critical distribution-transition. As a result of the distribution-transition, a high probability for one state is changed to another state.

Next, we show that the earlier transition caused by big noise is actually a "critical distribution-transition" in a stochastic system, that is, before the real bifurcation value ($p_c = 2$ of the deterministic system in the above example), where the probability distribution of the state drastically changes from one to the other in the form of the moments. Such a critical distribution-transition is related to the magnitude of noise and the distance between the parameter p and the bifurca-

tion value p_c, that is, the larger the noise is, the earlier the distribution-transition is; the nearer with the bifurcation value. Different from the traditional (critical) "state-transition" for a deterministic system, the critical "distribution-transition" for a stochastic system results in a new probability distribution. By such a transition, the probability of the current stable state may been significantly reduced while the probability of another stable state may be drastically increased.

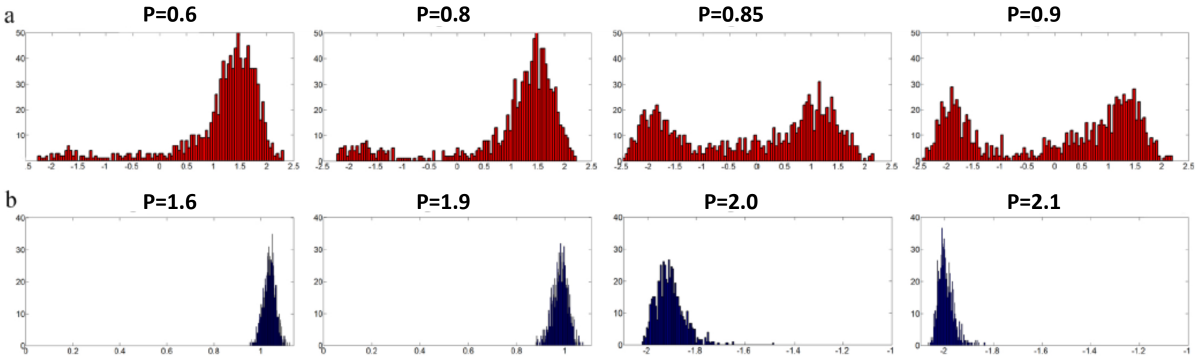

Figure S6: | **The changes in distribution of x in Eq.(S11) under a big noise and a small noise respectively. (a)**. The distributions of x under a big noise $\sigma = 1.5$, where the critical distribution transition is around $p = 0.8$. **(b)**. The distributions of x under a small noise $\sigma = 0.1$, where the critical distribution transition is around $p = 1.9$.

Specifically, based on the above example (with large samples), there is a probability distribution of state between $p = -2$ and $p = 2$ (see Fig.S5), during which the system may shift stochastically from a stable state to the other one. Figure S6 shows the changes of distribution of x in Eq.(S11) under a big noise $\sigma = 1.5$ (Fig.S6a) and a small noise $\sigma = 0.1$ (Fig.S6b) respectively. The drastic change of the distribution can be measured by the Kullback-Leibler (KL) divergence between two distributions, as shown in Fig.S7. It is worth noting that although the KL divergence can indicate the earlier transition due to the big noise, it requires many samples to estimate the distribution, which is not satisfied in many realistic cases.

From Fig.S6a, it can be seen that a significant change in the distribution (i.e., critical distribution transition) takes place after $p = 0.8$, which coincides with the Kullback-Leibler divergence index. (see the red curve in Fig.S7). Actually, under the noise $\sigma = 1.5$, the parameter

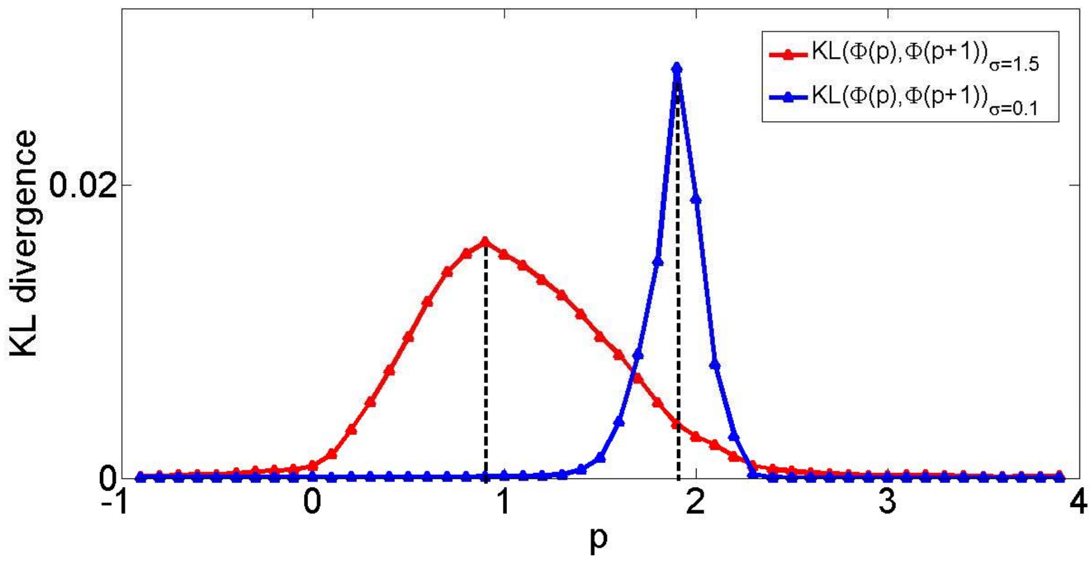

Figure S7: | **The Kullback-Leibler (KL) divergence curves of distributions respectively under a big noise and a small noise (a)**. Let $\Phi(p)$ denote the distribution of x when the parameter is p. The KL divergence $KL(\Phi(p), \Phi(p+1))$ is calculated and displayed to measure the critical distribution transition, where the red and blue curves respectively present the KL divergence of Φ under a big noise $\sigma = 1.5$, and under a small noise $\sigma = 0.1$. It can be seen that the red curve reaches the peak after $p = 0.8$ (the critical distribution-transition point for $\sigma = 1.5$), while the blue curve reaches the peak at $p = 1.9$ (the critical distribution-transition point for $\sigma = 0.1$), which coincides with the distribution-transition phenomena shown in the main text.

$p = 0.70191$ is a bifurcation point of 2-dimensional system (S14, S15) without noise, or the moment-system. Therefore, the parameter value at which the state of original system (1 dimensional) changes with a high probability, is approximately the bifurcation value of the expanded moment system (2 dimension) or the distribution-transition point.

B.3 Bifurcation point of the moment system corresponding to the critical distribution-transition moves earlier with the increase of noise level

Figure S8) shows the vector fields of the 2-dimensional moment system, where the equilibria merge to result in the bifurcations with the changing parameter for the cases of both big and small noises. In Fig. S8), the bifurcation point of the moment or distribution system indeed

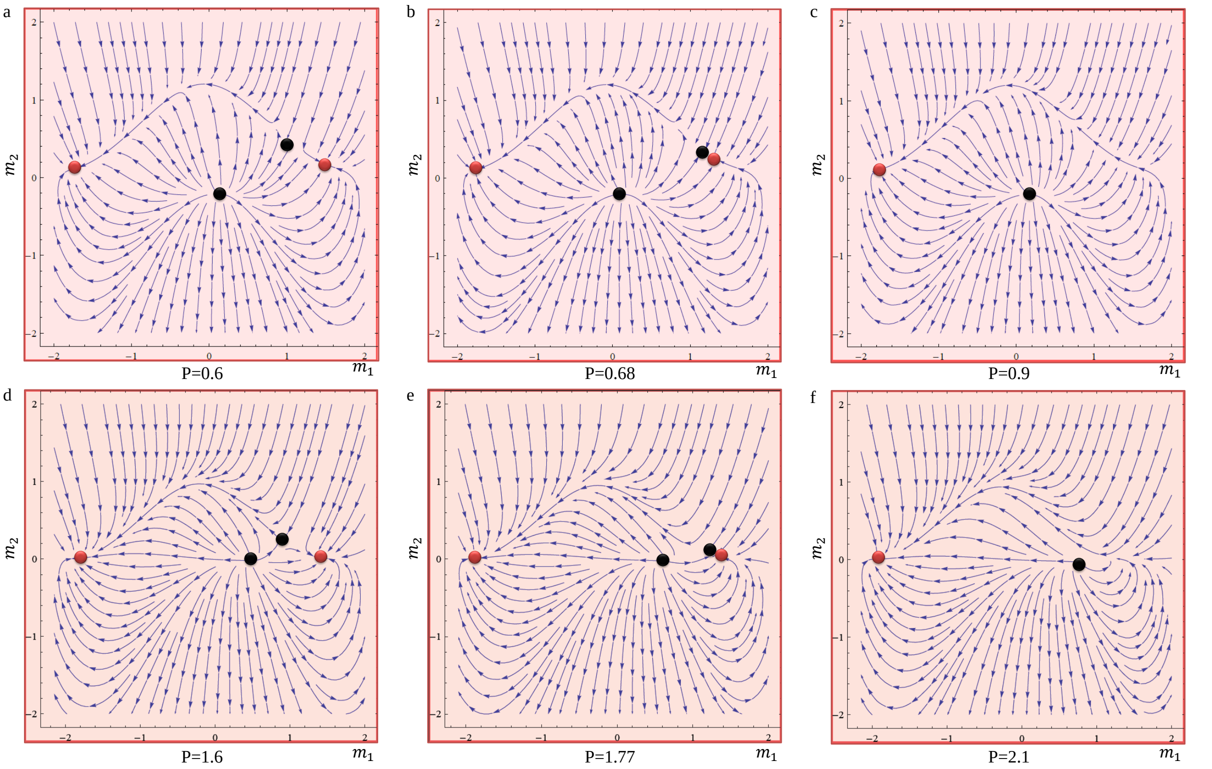

Figure S8: | **The vector fields of 2-dimensional moment system (S14, S15).** When $\sigma = 1.5$, **(a)**. the vector fields when $p = 0.6$. **(b)**. the vector fields when $p = 0.68$. **(c)**. the vector fields when $p = 0.9$. When $\sigma = 0.1$, **(d)**. the vector fields when $p = 1.6$. **(e)**. the vector fields when $p = 1.77$. **(f)**. the vector fields when $p = 2.1$. The red points represent the stable equilibria, and the black point is the unstable equilibrium. It can be seen that there is a bifurcation of the moment-system (S14, S15) between $p = 0.68$ and $p = 0.9$ (actually around $p = 0.80$) when $\sigma = 1.5$, and there is a bifurcation between $p = 1.77$ and $p = 2.1$ (actually around $p = 1.94$) when $\sigma = 0.1$. Clearly, the moment-system (S14, S15) is approximate to the original system, and thus its bifurcation point of the parameter (i.e., $p = 0.80$ for $\sigma = 1.5$ or $p = 1.94$ for $\sigma = 0.1$) is approximate to the critical distribution-transition point or the original stochastic system (i.e., $p = 0.8$ for $\sigma = 1.5$ or $p = 1.9$ for $\sigma = 0.1$), shown in Figs. S6-S7.

moves earlier with the increase of noise level. Thus, it validates the theoretical results, i.e., the critical transition occurs near the bifurcation point of the moment system rather than that of the original state system, but the bifurcation point of the moment system changes with the noise level. Thus, it is effective to detect the early-warning signals by the transformed data corresponding to the moment system.

C Numerical validation of DNM

C.1 Validation of DNM

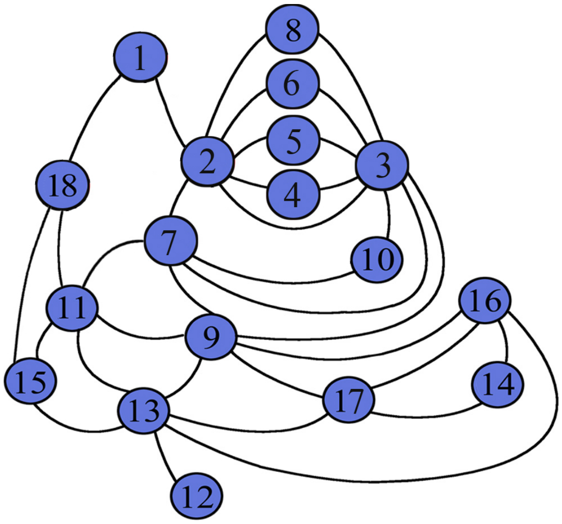

Figure S9: | **A model of a 18-molecular network.** In this sketch of a molecular network, there are 18 nodes whose dynamical regulatory relationships are given as stochastic system S16. The edges represent positive or negative regulations among nodes.

In this section, we use a regulatory network with 18 molecules (see Fig. S9) to conduct a numerical simulation and theoretically demonstrate the effectiveness of DNM for detecting the pre-transition state. Molecular networks are often used to study various biological processes such as transcription, translation, diffusion, and translocation processes that affect gene activities (*1, 4, 22–24*). The following 18 differential equations represent the gene regulation of 18 genes in a network where gene regulation is represented in a Michaelis-Menten form with the exception of the degradation rates, which are linearly proportional to the concentrations of the

corresponding genes.

$$\begin{cases}
\frac{\mathrm{d}x_1(t)}{\mathrm{d}t} = (\frac{-5P-1}{5} + \frac{3x_1(t)}{1+x_1(t)} + \frac{x_2(t)}{1+x_2(t)} - 100\left(\frac{x_1(t)}{1+x_1(t)}\right)^3 + \eta_1(t) \\
\frac{\mathrm{d}x_2(t)}{\mathrm{d}t} = \frac{(8-4P)x_3(t)}{15(1+x_3(t))} - 4\left(\frac{1+P}{15}\right)x_2(t) + \eta_2(t) \\
\frac{\mathrm{d}x_3(t)}{\mathrm{d}t} = \frac{(4-2P)x_2(t)}{15(1+x_1(t))} - 2\left(\frac{4+P}{15}\right)x_3(t) + \eta_3(t) \\
\frac{\mathrm{d}x_4(t)}{\mathrm{d}t} = \frac{4P-10}{15} + \frac{5-2P}{15(1+x_2(t))} + \frac{5-2P}{15(1+x_3(t))} - x_4(t) + \eta_4(t) \\
\frac{\mathrm{d}x_5(t)}{\mathrm{d}t} = \frac{(6-2P)x_2(t)}{15(1+x_1(t))} + \frac{(6-2P)x_3(t)}{15(1+x_3(t))} - \frac{6}{5}x_5(t) + \eta_5(t) \\
\frac{\mathrm{d}x_6(t)}{\mathrm{d}t} = \frac{4P-14}{15} + \frac{(7-2P)}{15(1+x_2(t))} + \frac{(7-2P)}{15(1+x_3(t))} - \frac{7}{5}x_6(t) + \eta_6(t) \\
\frac{\mathrm{d}x_7(t)}{\mathrm{d}t} = \frac{4P-16}{15} + \frac{2(4-P)}{15(1+x_2(t))} + \frac{2(4-P)}{15(1+x_3(t))} - \frac{8}{5}x_7(t) + \eta_7(t) \\
\frac{\mathrm{d}x_8(t)}{\mathrm{d}t} = \frac{(9-2P)x_2(t)}{15(1+x_2(t))} + \frac{(9-2P)x_3(t)}{15(1+x_3(t))} - \frac{9}{5}x_8(t) + \eta_8(t) \\
\frac{\mathrm{d}x_9(t)}{\mathrm{d}t} = -\frac{13}{15} + \frac{2}{15(1+x_2(t))} + \frac{2}{15(1+x_3(t))} + \frac{2}{5(1+x_7(t))} + \frac{2x_{11}(t)}{5(1+x_{11}(t))} + \frac{3x_{13}(t)}{5(1+x_{13}(t))} \\
\qquad + \frac{x_{16}(t)}{5(1+x_{16}(t))} + \frac{1}{5(1+x_{17}(t))} - 2x_9(t) + \eta_9(t) \\
\frac{\mathrm{d}x_{10}(t)}{\mathrm{d}t} = -1 + \frac{1}{5(1+x_2(t))} + \frac{1}{5(1+x_3(t))} + \frac{3}{5(1+x_7(t))} - \frac{11}{5}x_{10}(t) + \eta_{10}(t) \\
\frac{\mathrm{d}x_{11}(t)}{\mathrm{d}t} = \frac{3x_{13}(t)}{5(1+x_{13}(t))} - \frac{12}{5}x_{11}(t) + \eta_{11}(t) \\
\frac{\mathrm{d}x_{12}(t)}{\mathrm{d}t} = \frac{x_{13}(t)}{4(1+x_{13}(t))} - \frac{13}{5}x_{12}(t) + \eta_{12}(t) \\
\frac{\mathrm{d}x_{13}(t)}{\mathrm{d}t} = -\frac{2}{5} + \frac{2x_{16}(t)}{5(1+x_{16}(t))} + \frac{2}{5(1+x_{17}(t))} - \frac{14}{5}x_{13}(t) + \eta_{13}(t) \\
\frac{\mathrm{d}x_{14}(t)}{\mathrm{d}t} = \frac{6x_{17}(t)}{5(1+x_{17}(t))} - 3x_{14}(t) + \eta_{14}(t) \\
\frac{\mathrm{d}x_{15}(t)}{\mathrm{d}t} = -\frac{8}{5} + \frac{4}{5(1+x_{11}(t))} + \frac{4}{5(1+x_{13}(t))} - \frac{16}{5}x_{15}(t) + \eta_{15}(t) \\
\frac{\mathrm{d}x_{16}(t)}{\mathrm{d}t} = \frac{x_{17}(t)}{10(1+x_{17}(t))} - \frac{7}{2}x_{16}(t) + \eta_{16}(t) \\
\frac{\mathrm{d}x_{17}(t)}{\mathrm{d}t} = \frac{x_{16}(t)}{10(1+x_{16}(t))} - \frac{7}{2}x_{17}(t) + \eta_{17}(t) \\
\frac{\mathrm{d}x_{18}(t)}{\mathrm{d}t} = \frac{x_1(t)}{1+x_1(t)} + \frac{x_{11}(t)}{15(1+x_{11}(t))} + \frac{x_{15}(t)}{12(1+x_{15}(t))} - x_{18}(t) + \eta_{18}(t)
\end{cases} \tag{S16}$$

where P is a scalar control parameter ranging from -1 to 1 and $\eta_i(t)$ $(i = 1, 2, ..., 18)$ are Gaussian noises with zero means and covariances $\kappa_{ij} = \mathrm{Cov}(\eta_i, \eta_j)$. Here we set the amplitude κ_{ii} of η_i as 1. x_i $(i = 1, ..., 18)$ represent the concentrations of mRNA-i. There is a stable equilibrium when the parameter P is smaller than the critical value $P_c = 0$. When P passes $P_c = 0$, there is a state transition of the system (see Fig.3 in the main text). However, it is hardly to signal the transition based on the traditional method based on CSD due to the big noise. Therefore we carry out the DNM method by expanding the dimension of the system. The differential equations Eq.(S16) can be transformed into the difference equations $X(k+1) = f(X(k), P)$ using the Euler scheme (*25*), with a small time interval $\Delta t = 0.01$ when $P \in [-1, 1]$. Note that $X(k)$ is the vector of $X(t)$ at the time instant $k\Delta t$. By using this system,

we simulated the data and applied the DNM scheme to identify the pre-transition state as shown in Fig.3 in the main text. The simulations were performed in MATLAB(R2009a) using the Euler-Maruyama integration method with the Ito calculus (*25*).

C.2 Comparison with SVD

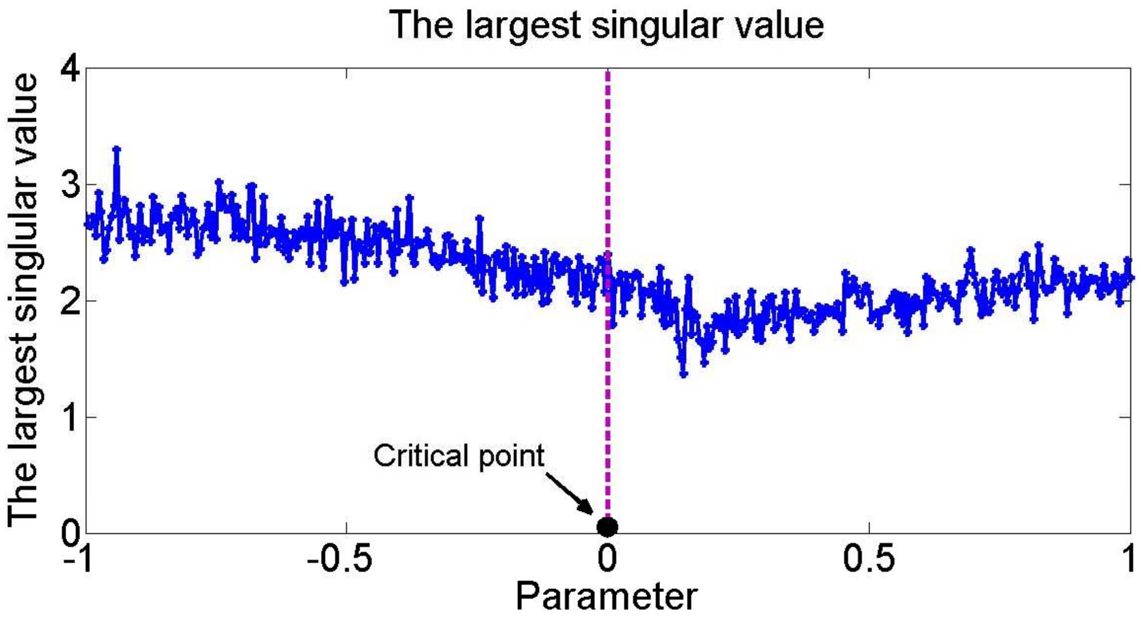

Figure S10: | **The change of the largest singular value.** This figure shows the change of the largest singular value when the system approaches the critical point. Clearly, due to noisy data and small number of samples, there is no significant signal near the critical point, i.e., the largest singular value obtained by SVD cannot indicate the imminent critical transition under big noise.

For the purpose of comparison to DNM, we also employed singular value decomposition to detect the early-warning signals of this multi-variable system from the observed data, i.e., using the largest singular value (Fig.S10), which is the square root of the dominant eigenvalue of MM^*, where M is the stochastically changing matrix of X in system (S16) and M^* is the conjugate transpose of M. As shown in Figure S10, due to noisy data and small number of samples, there is no significant signal for the largest singular value, which cannot indicate the

imminent critical transition under big noise.

D Algorithm for calculating DNM

For a given dataset, the calculation of DNM score is based on the following procedure.

Step 1 Dimension expanding.

At each time point, calculate the 1st and 2nd moments of the data for each variable.

Step 2 DNM scheme.

We conduct a new type of data normalization for all the variables obtained in **2**.

$$A = \frac{D_{\text{case}} - \text{mean}(N_{\text{control}})}{\text{SD}(N_{\text{control}})}, \tag{S17}$$

where A denotes the normalized expression data for each variable in each case sample, D_{case} is the data for each variable in every case sample, while the mean(N_{control}) and SD(N_{control}) are the mean and standard deviation for each variable in all the control samples, respectively.

At each sampling point (or period), by using the student t-test with significance level $p < 0.05$, choose those variables whose expressions show significant changes (in the sense of mean values) between the case samples and the control samples.

By using the false discovery rate (FDR), correct the multiple comparisons or multiple student t-tests for the variables selected in each period.

The two-fold change method is then adopted to further screen variables that exhibit relatively significant changes in standard deviation in each period.

Cluster variables at each sampling time point by correlations, i.e., each group (or the “in” group) is composed of molecules with high correlations, and the “out” group

contains all the other molecules. According to the theoretical results, if a time period is in or close to the pre-transition stage, then the clusters obtained in this period are potential dominant groups, and should satisfy the 3 criteria of DNM. Hence every cluster is a candidate of the DNM, whereas its corresponding period is a candidate of the transition point in the pre-transition state.

Step 3 Among all clusters, determine the dominant group or the DNM by significance analysis, i.e., the 3 criteria of DNM listed in main text are used to determine whether a candidate group is DNM.

Calculating the DNM score.

$$I = \mathrm{SD}\frac{\mathrm{PCC}_{in}}{\mathrm{PCC}_{out} + \epsilon}, \tag{S18}$$

where SD is the average standard deviation of all variables in DNM; PCC_{in} is the average Pearson's correlation coefficient between variables in DNM in absolute value; PCC_{out} is the average Pearson's correlation coefficient between a variable inside DNM and another one outside in absolute value; and ϵ is a small positive constant to avoid zero division. Here, instead of SD, it is also appropriate to use CV (coefficient of variation) in (S18) when the variables are not normalized.

We then screen clusters using the three criteria of DNM. That is, the SD of each variable in any candidate dominant group should sharply increases. The PCC$_{in}$ (in absolute value) for each pair of variables among any candidate dominant group should drastically increases. The PCC$_{out}$ between one variable in any candidate dominant group and another one outside should drastically be decreased.

E Application to three real datasets

For the three datasets, that is, gene expression profiling dataset of acute lung injury induced by phosgene gas and the ecological data of eutrophic lake state, we identified the pre-transition states by using DNM.

The gene expression profiling dataset of acute lung injury was downloaded from the NCBI GEO database (access ID: GSE2565, GSE13009, GSE30550) (www.ncbi.nlm.nih.gov/geo). In these datasets, probe sets without corresponding gene symbols were ignored during our analysis. The expression values of probe sets that are mapped to the same gene were averaged. The three datasets are described in Table S1. Besides, we described the data processing in details and conducted the functional analysis results (g:profiler: http://biit.cs.ut.ee/gprofiler/ and NOA: http://app.aporc.org/NOA/) (*29, 30*) for some important molecules.

Table S1: Descriptions of the three datasets

Experimental data	Description
Genomic data on lung injury due to carbonyl chloride inhalation exposure (GSE2565) (*33*)	
Sampling points	9 sampling points<br>0, 0.5, 1, 4, 8, 12, 24, 48, 72 (hours)
Number of observed objects	12,871 genes
Groups	control group and case group
Case data	6 subjects
Control data	6 control samples
Data for eutrophic lake (*34*)	
Sampling points	64 sampling points<br>From 1883 to 2009 (years)
Number of observations	6 indices
Groups	case group
Case data	1 subjects
Width of the sliding window	59 years
Data for bankruptcy (*35*)	
Number of observations	2 indices USD and EUR
Sampling points (EUR)	trading days from 12/01/1998 to 12/08/2011
Sampling points (USD)	trading days from 04/29/1999 to 06/06/2011

E.1 Dataset 1. Genomic data of the lung injury with carbonyl chloride inhalation exposure (i.e., acute lung injury)

This dataset was obtained in an experiment on toxic gas-induced lung injury effects, i.e., pulmonary edema (*33*), and was downloaded from the NCBI GEO database (ID: GSE2565) (www.ncbi.nlm.nih.gov/geo). In this data set, there are 22,690 original probe sets. We mapped them to the corresponding NCBI Entrez gene symbols by using the GEO annotation. Meanwhile, probe sets without corresponding gene symbols were not considered during our analysis. The expression values of probe sets mapped to the same gene were averaged. There were 12,871 genes left. Furthermore, the expression profiling information was mapped to the integrated networks, i.e., genes were linked and correlated by the combined functional couplings among them from various databases of protein-protein interactions. We downloaded the biomolecular interaction networks and functional linkage information for *Mus musculus* from various databases, including BioGrid (www.thebiogrid.org), STRING (http://string-db.org/), and KEGG (www.genome.jp/kegg). After removing the redundancy, we obtained 7,950 linkages in 6,683 mouse proteins/genes for acute lung injury. Next, the genes evaluated in these microarray datasets were mapped individually to these integrated functional linkage networks.

To study acute lung injury, a genomic approach was used to investigate the molecular mechanism of phosgene-induced lung injury. The experiments were conducted to determine the temporal effects of phosgene exposure on lung tissue antioxidant enzyme concentrations and the gene expression level, and these results were compared with those from air-exposed mice treated in a similar manner to assess the role of the GSH redox cycle in this oxidative lung injury model. To produce two groups of data, i.e., the control group data and case group data, two groups of CD-1 male mice were exposed to air or phosgene, respectively. Lung tissues were collected from air- or phosgene-exposed mice at 0.5, 1, 4, 8, 12, 24, 48, and 72 hr after exposure. The details of the experiment are available in the original paper (*33*). We introduce

some key background as well as our functional analysis as follows.

Phosgene gas, a nerve gas, is one of the most important and common chemical industry gases (*32*). Some pathogenic mechanisms of the acute lung injury induced by phosgene have been identified (*33*). According to the calculation results by the dynamical network marker (DNM), a major transition is considered to occur from 4 hr to 8 hr. Also, the pathway enrichment analysis and GO functional analysis showed that the identified genes in the DNM were closely related to the mechanism of disease progression (*33, 39*). Dysfunctions of glutathione metabolism and the chemokine signaling pathway related to inflammatory immune response were activated *in vivo*, which also implied the protection against the oxidant-like activity of phosgene. Pathways affected by the oxidant reaction became disordered, especially for signal transduction via protein-modified activation, such as the MAPK signaling pathway and Wnt signaling pathway. The decrease in the PH value induced by the HCl-release reaction affected some pathways that were sensitive to intracellular conditions and related to communication or transport channels, e.g., gap junctions. Some signaling pathways may also be relevant to repair, survival, disorders, and reproduction. The top significantly enriched signaling pathways are as follows: the Glutathione metabolism signaling pathway (with the significant P-value=9.6E-3) where glutathione is the main detoxifying agent in the body, and Glutathione deficiency contributes to oxidative stress, which plays a key role in aging and the pathogenesis of many diseases including cystic fibrosis, sickle cell anemia, etc. (*36*); the Mitogen-activated protein kinase (MAPK) signaling pathway (P-value=2.4E-2), and the abnormalities in MAPK signalling play a critical role in the development and progression of uncontrolled cell growth (*37*); the ErbB signaling pathway (P-value=3.8E-2), and the ErbB signaling pathway plays an important role during the growth and development of a number of organs including the central nervous system, which can be directly influenced by the nerve gas Phosgene (*38*); and Cytokine-cytokine receptor interaction (P-value=5.0E-2). Cytokines are soluble extracellular proteins or glycoproteins that are crucial

intercellular regulators and mobilizers of cells engaged in innate as well as adaptive inflammatory host defenses, cell growth, differentiation, cell death, angiogenesis, and development and repair processes aimed at the restoration of homeostasis. At the GO function level, some biological processes were also highly related to acute lung injury. For example, the expression profiles of some genes were related to abnormal changes in primary metabolic processes. Specifically, among the DNM members, *GCLC*, *gstA2*, *PGD*, *gsr* and *LOC630729* are involved in the glutamine-metabolic activities. Genes *FOS*, *DUSP1*, *Gadd45g*, *Hspb1*, *MYC*, *pla2g4a* and *IL1B* are related to the MAPK signaling pathway. This indicated the denaturation of lipoids, proteins, and nucleic acids that may have been oxidized by phosgene (*33, 39*).

Briefly, it was found that the main physiological effects occurred within the first 8 hours after exposure, resulting in common observations of enhanced BALF protein levels, increased pulmonary edema, and ultimately decreased survival rates (*33*). At the concentration delivered, 50%-60% mortality was routinely observed at 12 hours while 60%-70% mortality was observed at 24 hours (*33*). The detailed results are also available in the original paper (*33*). Early warning signals of lung injury based on the identified DNM are shown in Fig.4a of the main text, which showed that the pre-transition state may start around 4 hr, while the system may enter the after-transition state after 12 hr. Our prediction based on the DNM score agreed with the actual disease development.

To explain our method more clearly, we used acute lung injury as a concrete example to describe our computational procedure step by step. In GSE2565 data set, there are 22,690 original probe sets. We mapped them to the corresponding NCBI Entrez gene symbols by using the GEO annotation. Meanwhile, we screened out all probe sets with incorrect corresponding gene symbols while probe sets that detected the same genes were combined using the averaging method. After this procedure, there were 12,871 genes left.

Step 1 Choose differential expression genes from the high-throughput gene data for acute lung

injury. At each sampling point (or period), there are 12,871 genes. Each gene has 6 case samples and 6 control samples. At the 0 h sampling point, the case samples are identical to the control samples.

At each sampling point, by using the student t-test with significance level $p < 0.05$ and the false discovery rate (FDR) $(p_i(k_i) < (k_i/controlsize(i)) \times 0.05)$ we screened out $A = [0, 53, 184, 1325, 1327, 738, 980, 1263, 915]$ differential expression molecules for 9 periods or time points, respectively.

Based on set A of the selected differential expression molecules, through two-fold change screening, we obtain $B = [0, 29, 72, 195, 269, 163, 173, 188, 176]$ genes respectively for the 9 sampling time points.

Step 2 We carried out the dimension expanding scheme, by calculating the 1st and 2nd moments of the data in each 2-sampling-points sliding-window.

We conducted the data normalization for all the variables (the 1st and 2nd moments).

$$A = \frac{D_{\text{case}} - \text{mean}(N_{\text{control}})}{\text{SD}(N_{\text{control}})}, \tag{S19}$$

where A denotes the normalized expression data for each variable in each case sample, D_{case} is the data for each variable in every case sample, while the mean(N_{control}) and SD(N_{control}) are the mean and standard deviation for each variable in all the control samples, respectively.

Cluster variables at each sampling time point by correlations. We got the candidate groups (the 2nd and 3rd group among all clustering groups).

Step 3 Among all clusters, determine the dominant group or the DNM by significance analysis.

Calculating the DNM score.

$$I = \text{SD}\frac{\text{PCC}_{in}}{\text{PCC}_{out} + \epsilon}. \tag{S20}$$

The first cluster which is qualified to show a critical transition is the 3rd group clustered in the 5th sampling point (8 h), containing 169 genes. The obtained DNM (or DNB: dynamical network biomarker) is consistent with the real experimental phenomenon, and the DNM index start increasing sharply from the 4th time period (4 h) and reach peaks in the 5th time period (8 h, i.e., the pre-transition, see Fig.4a in the main text). These indices show that the pre-transition state starts near the 4th time period (4 h), and the system transitions to another state after the 5th time period (8 h). Our early-warning signals are coincident with the actual disease development that the most prominent physiological effects occur within the first 8 h after exposure, resulting in pulmonary edema and ultimately reducing survival rates (see the original paper (*33*)). Based on the dynamical information of the network, we have graphically illustrated the dynamical changes in the overall mouse PPI network (see Fig.4b-e in the main text), from which it can be seen that a critical transition occurs around 8 h sampling point (during 4h-12h period).

In order to show the detailed progression of the DNM-related gene group as well as the whole molecular network along the sampling time series, we presented the dynamics of the whole mouse network in which the DNM-related genes were clustered in the upper-left corner (see Fig.S11) to compare the dynamical changes between the members of the DNM-related genes and other genes. From Fig.S11, it is obvious that the group of the DNM provides significant signals as the system approaches the critical point. Clearly, there is a drastic change in the DNM at 8 h in terms of expression variations and network structure.

We listed all of the 169 identified DNM members in the Supplementary Table 'Identified DNM members A'.

E.2 Dataset 2. Ecological data about the eutrophic lake state

The data record the historical changes in the Erhai Lake catchment system in Yunnan, China (*34*). The monitoring data mainly provide historical trends for lake water quality, and several

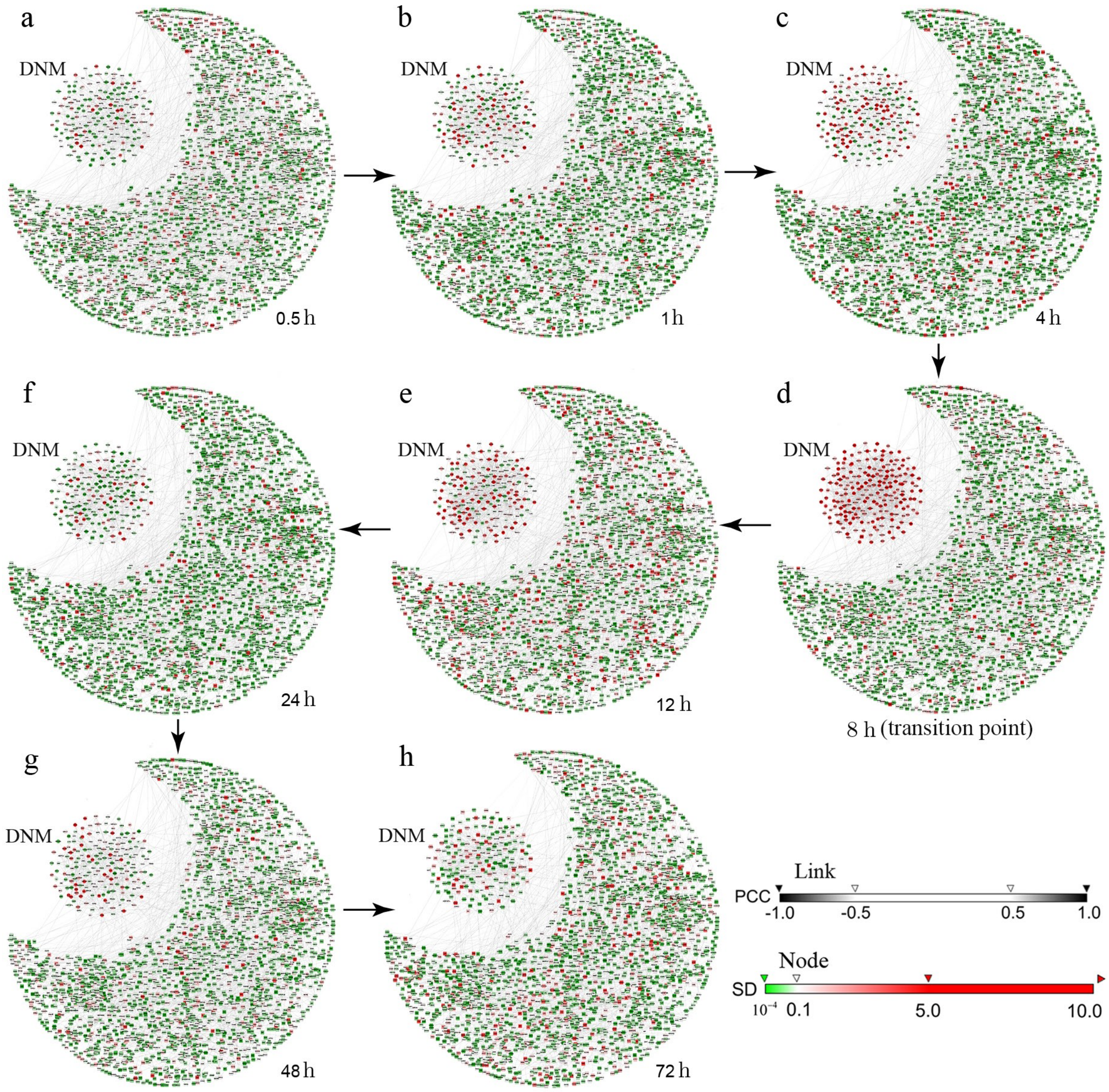

Figure S11: | **Dynamical changes in the network including the selected DNM during the progression of acute lung injury.** The figures show the dynamical changes of the molecular interaction network (protein-protein interactions and TF-target regulations) from **(a)** 0.5 hr, **(b)** 1 hr, **(c)** 4 hr, **(d)** 8 hr, **(e)** 12 hr, **(f)** 24 hr, **(g)** 48 hr to **(h)** 72 hr with the corresponding DNM, where the color of nodes represents the fluctuation strength in gene expressions, and each edge represents the correlations between two nodes. It can be seen that at 8 hr, there is a strong signal to indicate the pre-transition state (during 4h-12h).

related chemical indices. Microfossil and geochemical records from dated lake sediment cores were used to reconstruct the trends in the state of lake diatom communities and water quality

back to the 1880s, and these records seem to reproduce the abrupt change in algal states observed in recent monitored data, between 2001 and 2005. From the combined monitored and lake sediment data, it seems that a profound transition in the algal community occurred around 2001. From the original data, it is also pointed out that the transition in Erhai Lake in ∼2001 corresponds to the classic development of a bistable system (*34*), that is, the shift in the state of the diatom communities and the abrupt changes in other water quality indicators are consistent with the behaviour of a lake that is shifting from a stable state (i.e., the oligotrophic state) to an alternative stable state (the eutrophic state). Therefore, the DNM-based method is applicable to predict the imminent transition from one state to another (see Fig.4f in the main text). The dataset record the historical data for lake-water-quality variables including lake water level, DCA Axis 1 score and Hill's diversity N2; the concentrations of the related chemical indices including calcium (Ca), phosphorus (P), and abundance of nitrogen (N%) and carbon (C%); the climate indices including mean annual rainfall and temperature.

The identified DNM members of this ecological dataset include variables DCA Axis 1 score, N%, C%, the correlation between Hill diversity N2 and the concentration of phosphorus (P), the correlation between the concentrations of calcium (Ca) and the carbon (C%), and etc. Actually, the abundance of calcium in the sediment rose after 2001, as a result of the biologically induced precipitation of CaCO3 (calcite), which was linked to high rates of productivity by photosynthetic algae. The total sediment phosphorus abundance had a small rise after 2003, suggesting partial depletion of sediment phosphorus as a result of anoxic recycling. Sediment total organic carbon (C%) (the proportion of total sediment mass) and total nitrogen (N%) (the proportion of total sediment mass) abruptly rose after 2001, indicating rising levels of sedimenting organic matter as a result of the increased aquatic productivity (*34*).

We listed all of the 18 identified DNM members in the Supplementary Table 'Identified DNM members B'.

E.3 Dataset 3. Financial data about the bankruptcy of Lehman Brothers

The critical transitions in financial market are often referred to the broken of unstable "financial bubbles". In financial markets the participants slowly build up an ever densifying web of mutual dependencies through investments and transactions to hedge risks, which gradually create the unstable "bubbles" (*42, 43*). Detecting the onset of critical transitions in these complex dynamical systems is difficult due to the lack of the understanding of underlying mechanism as well as the insight to create models with predictive power.

Before declaring bankruptcy in 2008, Lehman Brothers was the fourth-largest investment bank in the United States, doing business in investment banking, equity and fixed-income sales and trading. On September 15, 2008, the firm filed for Chapter 11 bankruptcy protection following the massive exodus of most of its clients, drastic losses in its stock, and devaluation of its assets by credit rating agencies. Lehman's bankruptcy filing is the largest in US history, and is thought to have played a major role in the unfolding of the late-2000s global financial crisis.

The Financial data about the bankruptcy of Lehman Brothers consist of the daily prices of interest-rate swaps (IRS) in the USD and EUR currency. The data spans more than twelve years: the EUR data from 12/01/1998 to 12/08/2011 and the USD data from 04/29/1999 to 06/06/2011 (*35*). It is found that the CSD-based indices, i.e., SD and AR, "do not show a distinctive change of behavior around the time of the bankruptcy in both USD and EUR markets (*35*)" (also see Fig.S12). However, the DNM-based method provides the obvious signal for the critical transition (see Fig.4g in the main text). The DNM members of this financial dataset include the mean of USD (mean(USD)), the standard deviation of USD (SD(USD)), the mean of EUR (mean(EUR)), the standard deviation of EUR (SD(EUR)), and the correlation between USD and EUR (Corr(USD, EUR)). In the USD market, a long-term build-up of stress started in the beginning of 2004 and continued for more than four years, eventually peaking shortly before the bankruptcy (*35*). This is coincident with the common sense that markets created

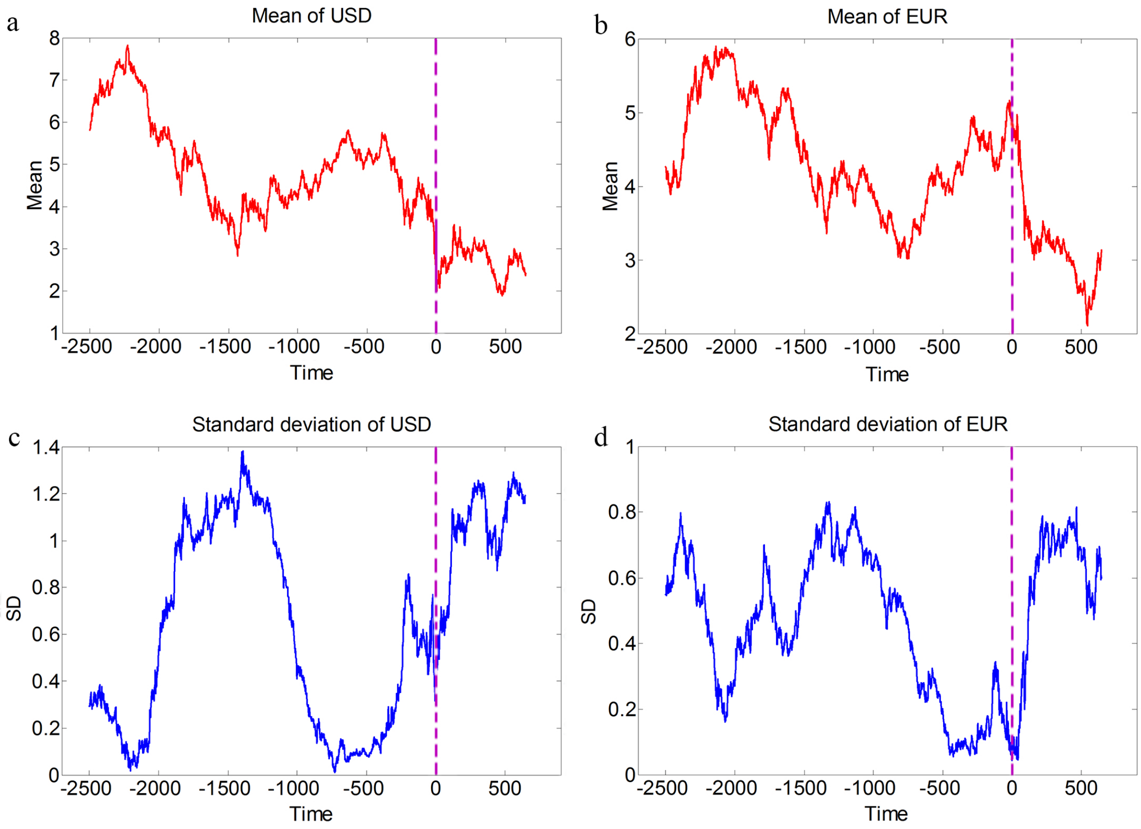

Figure S12: | **A sketch for the financial data.** **(a)** shows the mean of USD currency. **(b)** shows the mean of EUR currency.**(c)** shows the standard deviation curve of USD currency. **(d)** shows the standard deviation curve of EUR currency. It can be seen that the CSD-based indicator fails to signal a distinctive change of behavior around the time of the bankruptcy (time point 0, i.e., 2008/9/15) in both USD and EUR markets.

'bubbles (*44*), which grow slowly along the time and may 'burst, leading to sudden regime shifts triggered by a catalyzing event. The EUR market, on the other hand, appears to have played a more submissive role. The information dissipation length indicator rose distinctly for about half a year before the Lehman Brothers bankruptcy and diminished in about the same amount of time (*35*).

We listed all of the 5 identified DNM members in the Supplementary Table 'Identified DNM members C'.

F State-transition with small noise and distribution-transition with big noise

Figure S13 illustrates the mathematical meaning of the critical state detected by dynamical network markers for a system with small or big noise. For a system with small noise, the critical point is the bifurcation point of the original state system, whereas for a system with big noise, the critical point is the bifurcation point of the moment-system, which corresponds to the probability distribution. Note that it is the probability distribution in the basin of the stable equilibrium rather than the whole space. The moment-system with moment closure is the approximation of the real probability distribution, or conditional probability distribution. Thus, for a system with big noise, the critical point is for the probability distribution of the state. Clearly, the distribution-transition is the generalization of the traditional state-transition. For instance, the bifurcation for a moment-system with the order-two moments, will result in the drastic change of the partial distribution for Gaussian part (or distortion of the Gaussian part) rather than the real distribution.

G Comparison of distribution embedding scheme with support vector machine

Each individual system has its specific critical transition, and thus detecting early-warning signals of the critical transition is a system or individual specific problem. Although detecting early-warning signals of the tipping point is not a typical classification problem, to compare with the machine learning methods we design such a computational problem for classifying the before-transition samples and the pre-transition samples. In particular, we compared the performance of our embedding scheme with a machine learning scheme, i.e., the support vector machine (SVM). The SVM maps the observed sample space to a higher dimension space

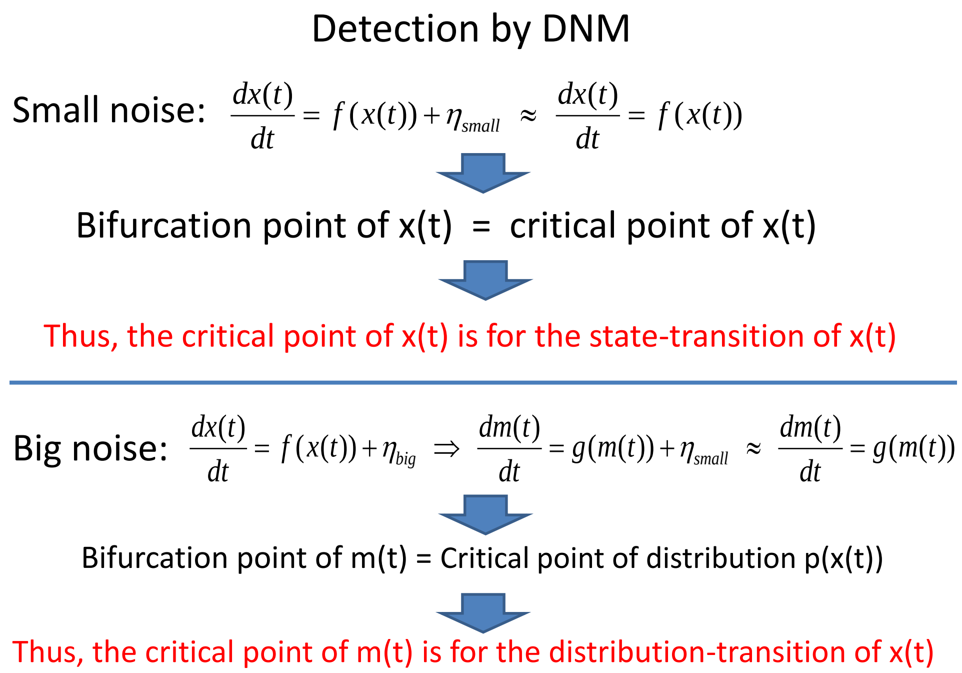

Figure S13: | **Detection of early-warning signals by dynamical network markers for a system with small or big noise.** For a system with small noise shown in the upper figure, DNM detects the critical state, which is the bifurcation point of the original deterministic system of $x(t)$. Thus, the critical state detected by DNM for $x(t)$ corresponds to the state-transition of $x(t)$. In other words, such a transition results in the drastic change of the state $x(t)$. On the other hand, for a system with big noise shown in the lower figure, there is no effective method to detect the critical state due to strong fluctuation. In this work, we transform the original system $x(t)$ with big noise to the moment system $m(t)$ with small noise, which can be detected by DNM for the critical state. Since the moment system corresponds to the probability distribution (i.e., a set of moments represent one probability distribution), the bifurcation point of the moment system is also the critical point of the distribution $p(x(t))$. Thus, the critical state of the moment system detected by DNM for $m(t)$ is the critical point of the distribution, or distribution-transition. In other words, such a transition results in the drastic change of the distribution of $x(t)$. Clearly, the distribution transition is the generalization of the state-transition, i.e., for small noise, they are equivalent.

and thus increases the dimensionality of the data, so that the samples can be classified by hyperplanes in the high-dimensional space. Thus SVM is a supervised learning scheme which increases the dimensionality of the data to improve the ability of the classifiers.

Specifically, we applied these two methods to a dataset from the eighteen-nodes network

Eq.(S16). Under a big noise $\sigma_i = \langle \eta_i^2 \rangle = 2, (i = 1, 2, ..., 18)$, we randomly chose 100 before-transition samples (under parameter $p = -1$), and 100 pre-transition samples (under parameter $p = -0.05$), based on which each scheme was applied to the classification. For SVM, 50 before-transition samples and 50 pre-transition samples are the training group, and the remaining samples are for testing. The classification model was constructed by using Gaussian kernel function. The ROC curves show in Fig.S14 that our distribution embedding scheme (AUC=0.8124) performs better than SVM (AUC=0.5988).

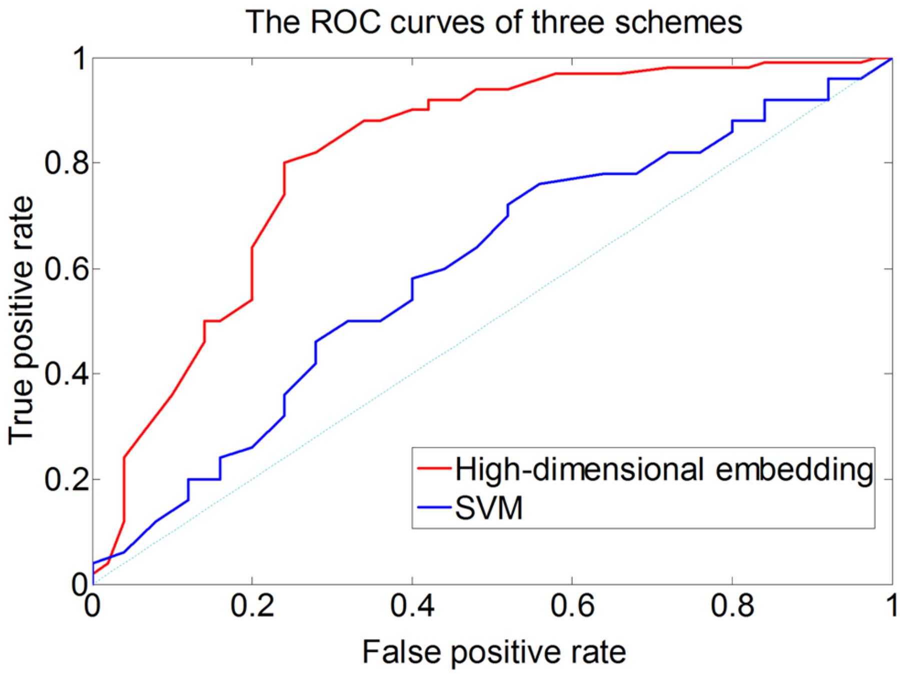

Figure S14: | **Comparison of distribution embedding scheme with a machine learning scheme.** Based on a dataset from the eighteen-nodes network Eq.(S16), the receiver operating characteristic (ROC) curves respectively show the performance of our distribution embedding scheme and that of the support vector machine (SVM). It can be seen that under big noise, the distribution embedding scheme or high-dimensional embedding (AUC=0.8124) works better than SVM (AUC=0.8124).

H The signal-to-noise ratio of the three datasets

We have investigated the signal-to-noise rate (SNR) by using the three real datasets at the critical points in terms of noises and signals. Specifically, we carried out the CSD-based method on each dataset with the original data (before the moment expansion). Then, we compared the signals between the CSD-method and our moment-expanding algorithm. The comparison of SNR is also presented in Table.S2. The signal-to-noise ratio (SNR) is calculated by the following formula:

$$SNR = \frac{\mu}{\sigma}$$

where μ is the signal mean and σ is the standard deviation of the noise.

Table S2. The signal-to-noise ratio (SNR) for the three real datasets.

	Signal-to-noise ratio (SNR)		
Datasets	Genomic data on lung injury induced by phosgene	Ecological data on a eutrophic lake	Financial data on the bankruptcy of Lehman Brothers
Before moment expanding	4.45	2.59	3.55
After moment expanding	6.50	16.57	9.96

The results indicated that SNRs in the higher dimensional space all increased after the implementation of the moment expansions, compared with those in the original space, which show the effectiveness of our method.

References

1. Chen, L., Wang, R., Li, C. & Aihara, K. *Modeling Biomolecular Networks in Cells: Structures and Dynamics*, (Springer, New York, 2010).

2. Voit, E.O. A systems-theoretical framework for health and disease: Inflammation and preconditioning from an abstract modeling point of view. *Math. Biosci.* **217**, 11-18 (2009).

3. Hovinen, E., Kekki, M. & Kuikka, S. A theory to the stochastic dynamic model building for chronic progressive disease processes with an application to chronic gastritis. *J. Theor. Biol.* **57**, 131-152 (1976).

4. Chen, L., Wang, R. & Zhang, X. *Biomolecular Networks: Methods and Applications in Systems Biology*, (John Wiley & Sons, Hoboken, New Jersey, 2009).

5. Guckenheimer, J. & Holmes, P. *Nonlinear Oscillations, Dynamical Systems, and Bifurcations of Vector Fields*, (Springer, 1983).

6. Arnol'd, V.I. *Dynamical systems V: bifurcation theory and catastrophe theory*, (Springer, 1994).

7. Murdock, J. *Normal forms and unfoldings for local dynamical systems*, (Springer, 2003).

8. Wiggins, S. *Global bifurcations and chaos: analytical methods*, (Springer, 1988).

9. Mlodinow, L. *The Drunkard's Walk*, (New York: Random House, 2008).

10. Cover, T. & Thomas, J. *Elements of information theory*, (Wiley, New Jersey, 2005).

11. Chen, L., Wang, R. Zhou, T. & Aihara, K. Noise-induced cooperative behavior in a multicell system. *Bioinformatics* **21**, 2722C2729 (2005).

12. Gillespie, C.S. Moment-closure approximations for mass-action models. *IET Syst. Biol.* **3**, 52C58 (2009).

13. Matis, T., Guardiola, I. Achieving Moment Closure through Cumulant Neglect. *The Mathematica Journal* **12**, (2010). doi:10.3888/tmj.12-2

14. Strogatz, S. H. *Nonlinear Dynamics And Chaos: With Applications To Physics, Biology, Chemistry And Engineering*, (Addison-Wesley, Reading, MA, 1994).

15. Chen,L. *et al.* Detecting early-warning signals for sudden deterioration of complex diseases by dynamical network biomarkers. *Scientific Reports* **2**(342), 1-8 (2012).

16. Strogatz, S. H. *Nonlinear dynamics and chaos: with applications to physics, biology, chemistry and engineering*. (Addison-Wesley, Reading, Massachussets, 1994).

17. Liu,R. *et al.* Identifying critical transitions and their leading networks for complex diseases. *Scientific Reports* **2**(813), 1-9 (2012).

18. Liu,R. *et al.* Identifying critical transitions of complex diseases based on a single sample. *Bioinformatics* **30**(11), 1579-1586 (2014).

19. Scheffer, M. *et al.* Early-warning signals for critical transitions. *Nature* **461**, 53-59 (2009).

20. Van Nes, E.H. & Scheffer, M. Slow recovery from perturbations as a generic indicator of a nearby catastrophic shift. *Am. Nat.* **169**, 738-747 (2007).

21. Wissel, C. A universal law of the characteristic return time near thresholds. *Oecologia* **65**, 101-107 (1984).

22. Becskei, A. & Serrano, L. Engineering stability in gene networks by autoregulation. *Nature* **405**, 590-593 (2000).

23. Chen, L. & Aihara, K. Stability of genetic regulatory networks with time delay. *IEEE Trans. Circuits Syst. I* **49**, 602-608 (2002).

24. Li, C., Chen, L. & Aihara, K. Stability of genetic networks with SUM regulatory logic: Lur'e system and LMI approach. *IEEE Trans. Circuits Syst. I* **53**, 2451-2458 (2006).

25. Kloeden, P. & Platen, E. *Numerical Solution of Stochastic Differential Equations*, (Springer, 1999).

26. Kolassa, John E. & Peter McCullagh. Edgeworth series for lattice distributions. *The Annals of Statistics* **18**, 981-985 (1990).

27. Barzel, & Biham, O. Binomial Moment Equations for Stochastic Reaction Systems. *Phys. Rev. Lett.* **106**, 150602 (2011).

28. Rencher, A. C. *Methods of Multivariate Analysis*, (Wiley, New York, 1995).

29. Reimand, J., Arak, T. & Vilo, J. g:Profiler – a web server for functional interpretation of gene lists (2011 update), *Nucleic Acids Res* **39**, W307-315 (2011).

30. Wang, J. *et al.* NOA: a novel Network Ontology Analysis method. *Nucleic Acids Res.* **39**, e87 (2011).

31. Gene cards: http://www.genecards.org/.

32. Wolfgang, S. & Werner, D. *"Phosgene" in Ullmann's Encyclopedia of Industrial Chemistry Wiley-VCH*, (Weinheim, 2002).

33. Sciuto, A. M., *et al.* Genomic analysis of murine pulmonary tissue following carbonyl chloride inhalation. *Chem. Res. Toxicol.* **18**, 1654-1660 (2005).

34. Wang, R., Dearing, J.A., Langdon, P.G., Zhang, E., Yang, X., Dakos, V. & Scheffer. M. Flickering gives early warning signals of a critical transition to a eutrophic lake state. *Nature* **492**, 419-422 (2012).

35. Quax, R., Kandhai, D. & Sloot, P. Information dissipation as an early-warning signal for the Lehman Brothers collapse in financial time series. *Scientific Reports* **3**, 1-7 (2013).

36. Wu, G., Fang, Y. Z., Yang, S., Lupton, J. R., & Turner, N. D. Glutathione metabolism and its implications for health. *The Journal of nutrition* **3**(134), 489-492 (2004).

37. Kim, E. K. & Choi, E. J. Pathological roles of MAPK signaling pathways in human diseases. *Biochimica et Biophysica Acta (BBA)-Molecular Basis of Disease* **4**(1802), 396-405 (2010).

38. Alroy, I. & Yarden, Y. The ErbB signaling network in embryogenesis and oncogenesis: signal diversification through combinatorial ligand-receptor interactions. *FEBS Lett.* **1**(410), 83-86 (1997).

39. Wang, P. *et al.* Mechanism of acute lung injury due to phosgene exposition and its protection by cafeic acid phenethyl ester in the rat. *Exp. Toxicol Pathol.* **24** (2011).

40. Klimenko A.Y. & Bilger W. Conditional moment closure for turbulent combustion. *Progress in Energy and Combustion Science* **25**, 595C687 (1999).

41. Majda A.J. & Kramer P.R. Simplified models for turbulent diffusion: Theory, numerical modelling, and physical phenomena. *Physics Reports* **314**, 237-574 (1999).

42. May, R. M. & Arinaminpathy, N. Systemic risk: the dynamics of model banking systems. J. R. Soc. Interface **7**, 823C838 (2010).

43. Haldane, A. G. & May, R. M. Systemic risk in banking ecosystems. *Nature* **469**, 351C355 (2011).

44. Sornette, D. Why Stock Markets Crash: Critical Events in Complex Financial Systems. (Princeton University Press, 2004).

Supplementary Table 'Identified DNM members'

A. Acute lung injury

DNM members	p-value	SD
Hsp90ab1	2.38E-10	0.04087
Slc2a1	1.13E-10	0.092623
Pdk2	1.60E-10	0.020142
Ptgs1	1.30E-09	0.069366
Ptpn14	1.08E-11	0.078193
Klf6	4.67E-07	0.074441
Serpina3n	7.99E-10	0.26976
Hes6	3.35E-08	0.029251
Als2cr13	5.66E-12	0.049826
Rnf5	1.14E-08	0.024356
Zfp36	1.28E-10	0.124548
Thbs1	6.32E-11	0.381639
Higd2a	3.42E-11	0.023156
Cxcr7	4.83E-07	0.072613
Klf4	4.60E-10	0.049678
Dhx32	2.01E-09	0.05761
Cyr61	3.71E-08	0.396
Hspd1	1.51E-08	0.178144
Loc100046232	3.96E-09	0.197075
Nmd3	1.33E-10	0.087827
Tob2	2.31E-09	0.047719
Psma5	1.77E-08	0.077423
Srxn1_Blvrb	0.006566	0.38397
Srxn1_Slc2a1	3.40E-11	0.185212
Srxn1_Dhx32	1.46E-09	0.22732
Srxn1_Adamts4	0.012015	0.180437
Esd_Ldlr	0.000162	0.570045
Esd_Cxcl16	1.60E-05	0.237233
Esd_Med8	0.034733	0.235243
Esd_Fos	4.80E-05	0.123527
Gclc_Eif6	1.00E-05	0.232833
Gjb3_Klhdc8a	1.00E-06	0.237048
Gjb3_Ctps	2.70E-05	0.485797
Gjb3_Irs2	2.00E-06	0.206056
Gjb3_Pla2g4a	5.00E-05	0.327429
Gjb3_Sult1a1	0.000119	0.244444
Gjb3_Selm	1.00E-06	0.388827
Gsta2_Dctpp1	1.10E-05	0.356852
Pgd_Med8	0.000688	0.344615
Ppl_Prdx1	1.76E-11	0.245119
Tinagl1_Il1b	5.90E-05	0.257371
Fgfbp1_Igj	0.000172	0.189926
Hsp90ab1_Spcs3	4.50E-05	0.441399
Hsp90ab1_Kti12	0.021878	0.863316
Abcc1_Loc100047896	1.64E-11	0.229009
Blvrb_Slc2a1	0.000132	0.420818
Blvrb_Tiparp	2.00E-06	0.337931
Blvrb_Tlk2	0.002276	0.835746
Blvrb_Dhx32	0.00155	0.43102
Blvrb_Adamts4	0.000101	0.378582
Gsr_Mtch1	0.001403	0.407237
Gch1_Tnfsf12	0.006268	0.320648
Mt2_Plscr2	2.90E-05	0.408598

Hspb1_Klf6	0.012551	0.181015
Hspb1_Cxcl16	0.000477	0.315647
Hspb1_Thbs1	2.40E-05	0.371511
Hspb1_Loc640441	1.06E-08	0.231753
Hspb1_Klf4	9.35E-09	0.273751
Hspb1_Fos	5.00E-04	0.189043
Slc2a1_Ptpn14	3.55E-09	0.170878
Slc2a1_Rnf5	3.00E-06	0.176245
Mal_Ppa1	2.00E-06	0.310943
Mal_Als2cr13	8.00E-06	0.375288
Pdk2_Loc630729	4.00E-05	0.405232
Pdk2_Tubb2c	2.44E-07	0.1614
Ptgs1_Med8	5.80E-05	0.380394
Lama3_S100a9	0.00011	0.066927
Lama3_Arcn1	8.60E-05	0.458731
Ifrd1_Ccl2	1.00E-06	0.292758
Gadd45g_Psmc1	0.002211	0.227541
Tnfsf12_Hes6	0.005493	0.171264
Hspa9_Cdh2	3.72E-10	0.245311
Fabp4_Kti12	2.70E-05	0.277604
Taldo1_Cyr61	0.000508	0.239241
Prss22_Nupr1	0.00357	0.339711
Afp_Lif	0.00022	0.424778
Ensmusg00000040078_Arcn1	0.000139	0.435126
Ppa1_Ctps	1.00E-05	0.507297
Ppa1_Areg	8.02E-11	0.120162
Ppa1_Cxcl16	0.000717	0.262089
Ldlr_Cxcr7	1.20E-05	0.440076
Ldlr_Nmd3	5.57E-09	0.224004
Eg383901_Timp1	0.006961	0.080515
Nupr1_Areg	8.79E-11	0.190037
Nupr1_Cxcl16	5.00E-06	0.305527
Nupr1_Thbs1	0.00013	0.279737
Nupr1_Ccrn4l	7.89E-08	0.192359
Nupr1_Edn1	2.70E-05	0.193538
Nupr1_Clu	0.000628	0.110055
Loc630729_Hsbp1	0.030719	0.341359
Loc630729_Hs6st1	0.010106	0.269517
Pkp3_Prdx1	4.80E-05	0.318972
Hspb8_Tle6	1.54E-09	0.184779
Klhdc8a_Gp49a	0.000283	0.215621
Klhdc8a_Rhoj	0.00031	0.402665
Enc1_Kti12	0.003057	0.527733
Errfi1_Plscr2	0.001115	0.374796
Errfi1_Arcn1	0.000169	0.350736
Myc_Lif	0.001152	0.63458
Ctps_Hbegf	6.10E-05	0.358457
Ctps_Prkci	0.000242	0.344347
Ctps_Sult1a1	2.90E-05	0.289255
Ctps_Scel	1.80E-05	0.342057
Serpina3n_Arcn1	0.000102	0.369235
Hes6_Eif1a	0.018146	0.055822
Hes6_Notch2	0.000538	0.17319
Loc100047868_Phldb1	5.80E-05	0.258957

Loc100047868_Sri	0.002699	0.164706
Maff_Lif	0.000294	0.585834
Maff_Epb4.1l5	0.005843	0.296004
Pde4b_Arcn1	8.30E-05	0.40299
Areg_Med8	2.60E-05	0.432661
Kctd9_Btg2	0.000569	0.195706
Als2cr13_Tbc1d15	0.000256	0.336779
Als2cr13_Tnpo3	0.019578	0.387089
Als2cr13_Gca	2.00E-06	0.441627
Myo5a_Trib3	0.003186	0.790796
Rnf5_Zfp36	2.00E-06	0.223092
Tmem49_Ogfrl1	0.014663	0.373819
Cxcl16_Med8	0.000157	0.458653
Cxcl16_Hspd1	1.50E-05	0.253252
Cxcl16_Eif6	0.000448	0.26819
Plaa_Ogfrl1	3.10E-05	0.164674
Gp49a_Plscr2	0.000445	0.564678
Gp49a_Tob2	9.30E-05	0.307533
Gtlf3b_Btg2	7.00E-06	0.354595
Zfp36_Taz	2.60E-05	0.425127
Thbs1_Med8	0.001778	0.379517
Thbs1_Dcbld1	0.000411	0.451862
Prdx1_Ccl2	0.00272	0.422894
Prdx1_Ddi2	0.012893	0.534472
Higd2a_Dusp1	8.90E-09	0.052136
Ereg_Btg2	5.78E-10	0.236091
Dnajc5_Tle6	1.00E-06	0.311395
Dnajc5_Btg2	1.26E-07	0.222343
Cxcr7_Rhoj	2.94E-10	0.210224
Rtkn2_Kti12	6.90E-05	0.356634
Litaf_Med8	0.001784	0.302859
Timp1_Med8	2.76E-08	0.237886
Loc640441_Med8	0.000393	0.329043
Loc640441_Dcbld1	3.00E-06	0.322184
Macrod1_Rnasek	6.40E-05	0.169429
Macrod1_Uhrf1bp1l	0.00077	0.220277
Bach2_Socs3	0.00137	0.264381
Bach2_Tuft1	9.83E-11	0.26052
Ccl2_Csf3r	0.000819	0.239654
Ccl2_Usp7	0.000652	0.446793
Ccl2_Psma5	1.25E-11	0.266345
Kti12_Arf3	0.000224	0.533188
Klf4_Med8	4.50E-05	0.381146
Klf4_Dcbld1	2.00E-06	0.319777
Loc672215_Lif	0.001623	0.436325
Ddi2_Usp7	0.009066	0.537159
Ddi2_C4b	5.99E-08	0.192083
Slc25a29_Btg2	1.70E-05	0.230468
Slc25a29_Arcn1	2.30E-05	0.460651
Prr13_Med8	0.002593	0.57498
Ogfrl1_Dctpp1	0.000263	0.440491
C1r_Lif	1.30E-05	0.410423
Usp7_Igj	0.001093	0.191992
Med8_Hspd1	0.00529	0.16984

Med8_Edn1	0.0044	0.350869
Med8_Fos	0.000259	0.421963
Sult1a1_Taz	0.001055	0.58253
Loc100046232_Eif6	7.00E-06	0.305779
Lif_Lace1	0.004105	0.497034
Lif_Plk3	1.70E-05	0.29693
Lif_Ch25h	0.004751	0.413246
Taz_Gtf2f2	1.21E-07	0.280002

B. The eutrophic lake

DNM members	p-value	SD
DCA Axis 1 score	1.67E-51	0.002991
N%	1.63E-46	0.018682
C%	1.54E-37	0.012677
Hill diversity N2_P	1.55E-41	0.095036
Hill diversity N2_C%	1.32E-37	0.145464
Hill diversity N2_N%	4.01E-30	0.18449
Ca_C%	1.07E-15	0.024797
P_N%	1.72E-31	0.582127
N%_C%	0.055919	0.009049
P_C%	1.94E-22	0.340956
Hill diversity N2_Ca	1.02E-36	0.104528
DCA Axis 1 score_Ca	9.98E-39	0.036262
Ca_N%	2.05E-15	0.060886
Ca_P	4.23E-33	0.064039
Ca	4.67E-24	0.006915
P	1.13E-27	0.015352
DCA Axis 1 score_Hill diversity N	6.22E-18	0.065883
DCA Axis 1 score_P	4.12E-38	0.129782

C. The bankruptcy of Lehman Brothers

DNM members	p-value	SD
USD	3.44E-25	0.0479
USD_USD	2.72E-09	0.1088
EUR	8.59E-15	0.0072
EUR_EUR	7.24E-24	0.1665
USD_EUR	7.74E-09	0.3085
